# *Candida albicans* Biofilm-Derived Extracellular Vesicles Are Involved in the Tolerance to Caspofungin, Biofilm Detachment, and Fungal Proteolytic Activity

**DOI:** 10.3390/jof9111078

**Published:** 2023-11-04

**Authors:** Justyna Karkowska-Kuleta, Kamila Kulig, Grazyna Bras, Karolina Stelmaszczyk, Magdalena Surowiec, Andrzej Kozik, Elzbieta Karnas, Olga Barczyk-Woznicka, Ewa Zuba-Surma, Elzbieta Pyza, Maria Rapala-Kozik

**Affiliations:** 1 Department of Comparative Biochemistry and Bioanalytics, Faculty of Biochemistry, Biophysics and Biotechnology, Jagiellonian University, Gronostajowa 7, 30-387 Kraków, Poland; 2 Doctoral School of Exact and Natural Sciences, Faculty of Biochemistry, Biophysics and Biotechnology, Jagiellonian University, Gronostajowa 7, 30-387 Kraków, Poland; 3 Department of Analytical Biochemistry, Faculty of Biochemistry, Biophysics and Biotechnology, Jagiellonian University, Gronostajowa 7, 30-387 Kraków, Poland; 4 Department of Cell Biology, Faculty of Biochemistry, Biophysics and Biotechnology, Jagiellonian University, Gronostajowa 7, 30-387 Kraków, Poland; 5 Department of Cell Biology and Imaging, Institute of Zoology and Biomedical Research, Jagiellonian University, Gronostajowa 9, 30-387 Kraków, Poland

**Keywords:** extracellular vesicles, biofilm, secreted aspartic proteinase, kininogen, antimicrobial peptide, caspofungin, *Candida albicans*, candidiasis

## Abstract

It has been repeatedly reported that the cells of organisms in all kingdoms of life produce nanometer-sized lipid membrane-enveloped extracellular vesicles (EVs), transporting and protecting various substances of cellular origin. While the composition of EVs produced by human pathogenic fungi has been studied in recent decades, another important challenge is the analysis of their functionality. Thus far, fungal EVs have been shown to play significant roles in intercellular communication, biofilm production, and modulation of host immune cell responses. In this study, we verified the involvement of biofilm-derived EVs produced by two different strains of *Candida albicans*—*C. albicans* SC5314 and 3147 (ATCC 10231)—in various aspects of biofilm function by examining its thickness, stability, metabolic activity, and cell viability in the presence of EVs and the antifungal drug caspofungin. Furthermore, the proteolytic activity against the kininogen-derived antimicrobial peptide NAT26 was confirmed by HPLC analysis for *C. albicans* EVs that are known to carry, among others, particular members of the secreted aspartic proteinases (Saps) family. In conclusion, EVs derived from *C. albicans* biofilms were shown to be involved in biofilm tolerance to caspofungin, biofilm detachment, and fungal proteolytic activity.

## 1. Introduction

For several representatives of the kingdom of fungi, the ability to produce extracellular vesicles (EVs) has been repeatedly reported in the last two decades, along with their compositional and structural characteristics. EVs are nanometer-sized, typically spherical structures of cellular origin, packed with plentiful and diversified cargo, and surrounded and protected by a lipid bilayer [1,2,3]. Although some assumptions about the role of EVs have already been explored or might be inferred based on the analysis of the vesicular content, the functionality of fungal EVs still requires thorough exploration and verification [4,5]. Recently, it has been established that fungal EVs can play important functions in intercellular, interspecies, and interkingdom communication, presumably using the different molecules they transport, that is, numerous proteins, peptides, nucleic acids, lipids, and other signaling compounds [6,7,8]. In the fungi pathogenic to humans, the EVs they produce have been frequently demonstrated to be involved in interactions with different types of host cells, thus strongly contributing to microbial virulence related to the ability to initiate infection and further dissemination within the human organism [9,10,11].

Currently, medically important fungi include several yeast species from the genus *Candida*, which are globally distributed and commonly identified as causative agents of both mild superficial infections and disseminated systemic diseases threatening human health, especially in immunocompromised individuals [12,13]. One of the essential mechanisms of candidal pathogenicity is the ability to produce biofilms formed by numerous cells of different lifespans and morphologies submerged in a dense biofilm matrix composed of polysaccharides, proteins, lipids, and nucleic acids. The biofilm lifestyle is accompanied by increased tolerance to host immunity and antifungal drugs, as well as the production of a wide arsenal of different virulence factors by fungi living in these organized, multicellular consortia [14,15,16]. Several studies have indicated the involvement of candidal EVs in the regulation of the biofilm life cycle, cellular morphology, biofilm matrix production, and tolerance to antifungal drugs. In particular, it has been previously shown that EVs produced by *C. albicans* yeast-like cells are involved in the maintenance of the fungal cell wall integrity [17]. At the same time, these EVs inhibit the morphological transition of yeast cells to hyphae and alter the formation of the *C. albicans* biofilm through the activity of different lipid compounds, i.e., sesquiterpenes, diterpenes, and fatty acids [18]. EVs produced by hyphae and biofilm cells were shown to be equipped with a higher number of virulence factors and to exert a more significant impact on host cells, contributing to the increased pathogenicity of fungi [17,18,19].

Extensive research on the effect of fungal vesicles on biofilm formation has also shown that EVs of various *Candida* species significantly participate in biofilm initiation, development, drug resistance, and biofilm–cell dispersion through the activity of a vesicular proteinaceous cargo containing enzymes involved in the production and rearrangement of polysaccharide matrix components [20,21,22]. One of the mechanisms of biofilm resistance to different antifungals is the sequestration of drugs by biofilm matrix containing β-glucans [23,24]. Therefore, vesicles involved in matrix production by providing both matrix components and essential enzymes can also contribute to reduced fungal susceptibility to antifungal treatment. Such a phenomenon has already been demonstrated for *Candida* biofilm-derived EVs that increase the tolerance of biofilm formed by five *Candida* species—*C. albicans*, *C. tropicalis*, *C. glabrata*, *C. parapsilosis*, and *C. auris*—to fluconazole [19,20,22]. Fluconazole is a fungistatic drug from the group of azoles, targeting cell membranes via inhibition of lanosterol-14α-demethylase, which is essential for ergosterol biosynthesis [25]. Another important antifungal drug, caspofungin, from the group of echinocandins, inhibits β-1,3-glucan synthase, altering the biogenesis of the fungal cell wall and components of the biofilm matrix [25]. Thus far, an increased biofilm tolerance for caspofungin mediated by fungal EVs has been demonstrated only for *C. tropicalis* [19].

In addition to the enzymes involved in the formation of the polysaccharide biofilm scaffold, *Candida* EVs also contain and transport a number of other enzymatic proteins, like several enzymes involved in basic intracellular metabolic pathways, as well as proteases, including those from the secreted aspartic proteinase (Saps) family [19,20,26]. *C. albicans* Saps comprise ten proteins (Sap1–Sap10) with different substrate specificity, expression profiles, and localization, which are extremely important for the physiology and virulence of *C. albicans* [27,28]. Also, in other studies on EVs produced by different species of pathogenic protozoa and bacteria, proteins with confirmed proteolytic activity were detected in microbial vesicles. Since proteinases are particularly important virulence factors for various infectious microorganisms, it is worth considering their activity as a part of the cargo of released EVs. EVs are structures that play an important role during infection and response to host immunity [29,30,31,32,33].

Therefore, in the current study, to broaden the picture of the functionality of *C. albicans* EVs derived from fungal biofilm, we examined their influence on the tolerance of *C. albicans* biofilm to caspofungin, investigated their effect on one of the mechanisms of spreading infection—biofilm detachment—and examined the proteolytic activity of EVs against host molecules. Furthermore, our research indicates some differences between the two investigated *C. albicans* strains.

## 2. Materials and Methods

### 2.1. Yeast Strains and Growth Conditions

*C. albicans* strains 3147 (ATCC 10231) and SC5314 were grown in 20 mL of liquid YPD medium (1% yeast extract, 2% soybean peptone, and 2% glucose, Sigma-Aldrich, St. Louis, MO, USA) at 30 °C for 18 h on an orbital rotary shaker MaxQ 6000 (170 rpm) (Thermo Fisher Scientific, Waltham, MA, USA). To estimate cell number, *C. albicans* cells were centrifuged for 5 min at 3000× *g*. The cell pellet was then suspended in sterile phosphate-buffered saline (PBS), pH 7.4 (Biowest, Nuaillé, France), and the optical density (OD) at 600 nm was measured in a glass cuvette with a 1 cm pathlength with the use of a Shimadzu UVmini-1240 spectrophotometer (Shimadzu, Kyoto, Japan). For a large-scale biofilm culture, *C. albicans* cells (1 × 10^8^) were inoculated into 100 mL of RPMI 1640 medium buffered with 25 mM HEPES, pH 7.3 ± 0.3 (Biowest) and cultured at 37 °C in sterile roller bottles (Corning Inc., New York, NY, USA) with a roller rack speed of 3 rpm for 48 h and medium exchange after 24 h.

### 2.2. Isolation of Extracellular Vesicles

*C. albicans* extracellular vesicles were isolated from the culture medium collected after biofilm growth as previously described [19] with some modifications. Briefly, supernatants were centrifuged twice at 4000× *g* for 15 min at 4 °C, each time discarding the cell pellet, and then concentrated using an Amicon Ultra-15 Centrifugal Filter Unit with a 100-kDa cutoff (Merck, Darmstadt, Germany) and filtered using an Ultrafree-CL Centrifugal Filter with a pore size of 0.65 µm (Merck). Ultracentrifugation of concentrated supernatant was performed at 4 °C for 1 h using a fixed-angle type 60 Ti Rotor in an Optima™ LE-80K Ultracentrifuge (Beckman Coulter, Brea, CA, USA) at a rotor speed of 45,000 rpm, corresponding to a relative centrifugal field of 144,000× *g* (k factor 112). After washing with PBS buffer once, the obtained EVs were transferred in 200 µL of PBS to Eppendorf tubes, aliquoted, and stored at −80 °C.

Protein concentration in the obtained fractions was estimated using *o*-phthalaldehyde (OPA; Sigma-Aldrich) modification of the primary amine groups of proteins. The fluorescence intensity measurements were performed with excitation and emission wavelengths of 340 and 455 nm, respectively, with a Synergy H1 microplate reader (BioTek Instruments, Winooski, VT, USA) using a black, flat-bottom 96-well microplate (Greiner Bio-One, Kremsmünster, Austria) as previously described [26].

### 2.3. TEM and NTA Analysis of Extracellular Vesicles

Transmission electron microscopy (TEM) was exploited for visualization of *C. albicans* EVs with the use of formvar-coated, 300-mesh copper grids and negative staining. EV samples were prepared with 2% uranyl acetate (Chemapol, Prague, Czech Republic). Then, imaging was performed using a JEOL JEM-2100 HT transmission electron microscope (JEOL, Tokyo, Japan) at an accelerating voltage of 80 kV. Images were taken using a 4 k × 4 k camera (TVIPS) equipped with the EMMENU software version 4.0.9.87. 

The measurements of size and concentration of *C. albicans* EVs were performed using the nanoparticle tracking analysis (NTA) and NanoSight NS300 system with camera type sCOS with laser Blue488 and NTA software Version 3.4 (Malvern Instruments, Malvern, UK) at 25 °C in PBS filtered through a 0.22 µm filter (Lonza, Basel, Switzerland). Each sample was recorded three times for 60 s with a camera level of 12 and a detection threshold parameter of 2. The size parameters of the EVs were measured; factors D10, D50, and D90 mean that 10%, 50%, and 90% of the EV population had a diameter of less than or equal to the presented value.

### 2.4. Identification of Vesicular Proteins Using Liquid Chromatography–Coupled Tandem Mass Spectrometry (LC–MS/MS)

The identification of proteins from biofilm-derived *C. albicans* EVs was performed using a similar procedure as previously described [19,34] with some modifications. Briefly, a total amount of EVs corresponding to 20 µg of protein (equivalent to 8 × 10^10^ and 5 × 10^10^ EVs for *C. albicans* 3147 and *C. albicans* SC5314, respectively) was suspended in 100 µL of 100 mM Tris-HCl buffer, pH 7.6, with 1% sodium dodecyl sulfate and sonicated in four cycles of 30 s with UP50H Compact Lab Homogenizer with amplitude 80%, cycle 0.5, 50 W, 30 kHz (Hielscher Ultrasonics, Teltow, Germany). Then, samples were vigorously shaken for 5 min at 95 °C and centrifuged for 12 min at 12,000× *g*. Next, the pellet was discarded, and the collected supernatant was precipitated by adding one volume of trichloroacetic acid to four volumes of the sample and incubated overnight at −20 °C. The mixtures were centrifuged at 10,000× *g* for 15 min at 10 °C, washed twice with ice-cold acetone, and the obtained pellet was dissolved in 100 µL of 10 mM HEPES buffer, pH 8.5. Further procedures were implemented strictly according to the protocols described by Surman et al. [35]. Briefly, paramagnetic bead technology based on single-pot solid phase-enhanced sample preparation was used for protein extraction with the use of two types of SpeedBeads—GE45152105050250 and GE65152105050250 (Sigma-Aldrich)—mixed in equal parts, followed by protein reduction with dithiothreitol, alkylation with iodoacetamide, and hydrolysis with 30 µL of 0.027 µg/µL Trypsin/Lys-C Mix (Promega, Mannheim, Germany). Obtained peptides were identified by MS/MS analysis using the UltiMate 3000 RSLCnano System coupled with Q Exactive mass spectrometer (Thermo Fisher Scientific) with a DPV-550 Digital PicoView nanospray source (New Objective, Woburn, MA, USA) after prior peptide separation on a trap column (Acclaim PepMap 100 C18, 75 μm × 20 mm, 3 μm particle, Thermo Fisher Scientific) and on an analytical column (Acclaim PepMap RSLC C18, 75 µm × 500 mm, 2 µm particle, Thermo Fisher Scientific). The Proteome Discoverer platform (v.1.4, Thermo Fisher Scientific) was used to process the obtained RAW files and proceeded to further search with an in-house MASCOT search engine (v.2.5.1, Matrix Science, London, UK). The Swiss-Prot protein sequence database was used with the following taxonomy restrictions: Fungi (number of sequences: 569,793; the number of sequences after taxonomy: 36,331). The following parameters were applied: fixed modification—cysteine carbamidomethylation; variable modifications—methionine oxidation; precursor mass tolerance—10 ppm; fragment mass tolerance—20 mmu. Target Decoy PSM Validator was used with the maximum false discovery rate (FDR) for peptides designated as 0.01. The mass spectrometry proteomics data have been deposited with the ProteomeXchange Consortium via the PRIDE partner repository [36], with the dataset identifier PXD044271.

### 2.5. Labelling of C. albicans EVs with CFSE

For the fluorescent labeling of *C. albicans* EVs, a reaction mixture was prepared in 100 µL of PBS containing 8 × 10^10^ EVs and 40 µM CellTrace™ CFSE (carboxyfluorescein succinimidyl ester) dye (Thermo Fisher Scientific) and incubated for 30 min at 37 °C in the dark, then the sample volume was adjusted to 500 µL with PBS. Separation by size exclusion chromatography was performed to remove excess dye from the sample with the use of qEVoriginal/70 nm columns (IZON Science, Christchurch, New Zealand) strictly according to the manufacturer’s instructions, and the obtained fractions containing EVs were combined. A mock chromatographic separation was also performed with a sample containing the dye alone, without EVs, to obtain control fractions for further measurements. The EV-labeling efficiency was verified by measuring the fluorescence intensity at excitation and emission wavelengths of 480 and 520 nm, respectively, with a Synergy H1 microplate reader using a black, flat bottom 96-well microplate (Greiner Bio-One), accompanied by simultaneous measurement of the absorbance of the same fractions at 280 nm in a glass cuvette with a 1 cm pathlength with a Shimadzu UVmini-1240 spectrophotometer. CFSE-labelled EVs were then aliquoted and stored at −80 °C for further use.

### 2.6. Examination of EV Proteolytic Activity

The verification of EV proteolytic activity was performed with the EnzChek™ Protease Assay Kit (Thermo Fisher Scientific) according to the manufacturer’s instructions. Briefly, *C. albicans* EVs (1 × 10^10^) were suspended in 100 µL of PBS, pH 7.4, with 1 μg of BODIPY FL casein, and incubated in the wells of a black, flat-bottom 96-well microplate (Greiner Bio-One) at 37 °C in the dark for 24 h. Before the action of proteinases, the fluorescence of the conjugate is almost totally quenched. After hydrolysis catalyzed by proteinases, a highly fluorescent dye is released, resulting in an increase in fluorescence intensity proportional to proteinase activity. Then, the fluorescence intensity was measured with excitation and emission wavelengths of 485 and 528 nm, respectively, with a Synergy H1 microplate reader.

### 2.7. Biofilm Formation in the Presence of EVs, Caspofungin, and Saps

**Figure 1:**
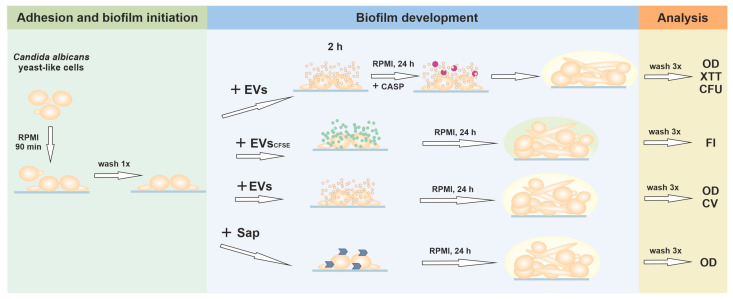
Diagram presenting the experimental variants performed in this study. EVs—extracellular vesicles; Sap—secreted aspartic proteinases; CASP—caspofungin; OD—optical density measurement; XTT—XTT reduction assay; CFU—colony forming units counting; FI—fluorescence intensity measurement; CV—crystal violet staining.

A graphical presentation of the experimental variants related to biofilm formation in the presence of different biofilm-affecting factors is presented in Figure 1.

In the first stage of biofilm formation, *C. albicans* cells (1 × 10^5^) were incubated in 100 µL of RPMI 1640 medium with phenol red, buffered with 25 mM HEPES, pH 7.3 ± 0.3 (Biowest) for 90 min at 37 °C in an atmosphere of 5% CO_2_ and 95% humidity in the wells of a Corning^®^ 96-well black/clear flat bottom polystyrene high bind microplate (Corning Inc., Corning, NY, USA). After that, the growth medium with non-adherent cells was removed, the wells were gently rinsed once with 100 µL PBS, and then 200 µL of RPMI 1640 medium buffered with 25 mM HEPES, pH 7.3 ± 0.3, were added to the adhered fungal germ tubes for further biofilm growth. Cells cultured for the next 24 h only in RPMI medium constituted a control biofilm, not subjected to any additional factors. In other approaches, the specified biofilm-affecting agents described below were additionally introduced for further incubation for certain time periods at 37 °C in an atmosphere of 5% CO_2_ and 95% humidity. After biofilm cultivation, the optical density (OD) of the biofilm in the microplate wells was measured at 600 nm with the nine-point area scan reading method before rinsing the biofilm. Then, the biofilm was gently washed three times with 200 µL PBS, and the OD measurement was repeated. The measured OD values obtained for microplate wells without biofilm were subtracted from the values measured for biofilm. After this step, further analyses of biofilms were additionally performed, as described in Section 2.8.

To observe the effect on biofilm tolerance to caspofungin (CASP) in the presence of EVs, after the first step of biofilm formation (cell adherence for 90 min as described above), 100 µL of RPMI medium containing 2 × 10^9^ EVs were added to the microplate wells for 2 h. Then, additional 100 µL of RPMI were added together with caspofungin (Sigma-Aldrich) to a final drug concentration of 0.005 µg/mL and a final medium volume of 200 µL. Microplate wells with cells with the same concentration of caspofungin in 200 µL of RPMI served as a control for the antifungal effect of the applied drug. The effect of EVs themselves on biofilm formation and detachment was tested by adding 200 µL of RPMI medium containing 2 × 10^9^ EVs after 90 min incubation and further cultivation for 24 h.

To verify the location of EVs within the formed fungal biofilm, 4 × 10^8^ CFSE-labeled EVs in 200 µL of RPMI medium without phenol red (Biowest) were added after 90 min of the first stage of biofilm formation to adhered germ tubes and incubated further for 24 h. After incubation, the biofilm was washed with 200 µL of PBS. The fluorescence intensity (FI) measurement was performed at excitation and emission wavelengths of 480 and 520 nm, respectively, with a Synergy H1 microplate reader The control consisted of *C. albicans* cells forming biofilm in the presence of a fraction obtained after the abovementioned mock chromatographic separation of CFSE dye using qEVoriginal columns; the additional control consisted of wells without fungal biofilms but incubated with labeled EVs under the same conditions and washed as described above. The RFU values for *C. albicans* 3147 biofilms alone and for empty wells incubated with CFSE-labeled EVs alone and rinsed have been subtracted from the values measured for biofilm.

The influence of Sap proteinases on biofilm detachment was verified after the addition of the appropriate recombinant *C. albicans* proteinase, obtained as previously described [37,38], at a concentration of 0.2 µg/mL in 200 µL of RPMI medium, to the adhered fungal germ tubes initiating biofilm as described above, and incubation further for 24 h.

### 2.8. Biofilm Analysis

The biofilm thickness was assessed with 0.5% crystal violet (CV) (Sigma-Aldrich) staining for 15 min, followed by washing the wells five times with 200 µL of water and subsequent destaining with 120 µL of 96% ethanol for 15 min. The measurement of absorbance at 590 nm was carried out after transferring 100 µL of the supernatant to a new 96-well microplate (Sarstedt, Nümbrecht, Germany). The measured absorbance values obtained for microplate wells without biofilm were subtracted from the values measured for the wells with biofilm.

The measurement of the metabolic activity of cells in the biofilm was performed with the XTT (sodium 3′-[1-(phenylaminocarbonyl)-3,4-tetrazolium]-bis (4-methoxy6-nitro) benzene sulfonic acid hydrate) (Thermo Fisher Scientific) test after the incubation for 40 min at 37 °C as described previously [19]. The measurement of absorbance at 450 nm was completed after transferring 100 µL of the supernatant to a new 96-well microplate (Sarstedt). The absorbance signal obtained for wells without biofilm but subjected to the same procedure was subtracted from the rest of the samples. Absorbance measurements were performed with a Synergy H1 microplate reader.

Additionally, from independent wells, biofilm-forming cells were mechanically removed in 50 µL of PBS, thoroughly vortexed, and after preparation of a ten-fold dilution series, 10 µL of cell suspensions were plated on YPD agar plates for CFU (colony forming unit) counting after incubation for 24 h at 30 °C.

### 2.9. Analysis of Peptide NAT26 Degradation

Kininogen-derived peptide NAT26 with the amino acid sequence of NATFYFKIDNVKKARVQVVAGKKYFI was purchased from Lipopharm (Zblewo, Poland). Samples for peptide degradation in the presence of *C. albicans* EVs were prepared and analyzed similarly as described previously [39,40] with some modifications. Briefly, a 100 µL mixture of NAT26 peptide (at a final concentration of 10 µM) and 4 × 10^9^ EVs was prepared in 20 mM phosphate buffer, pH 7.0, in Eppendorf tubes and incubated for 24 h at 37 °C. The peptide incubated only in the buffer served as a control. After incubation, samples were mixed with 20 μL of 1 M HCl, centrifuged at 13,000× *g* for 10 min, and the peptide-containing supernatants were passed through 0.22 μm filters (Sigma-Aldrich). Then, peptides were separated by high-performance liquid chromatography (HPLC) on a Eurosil Bioselect 300-5 C-18 HPLC column (5 μm, 4 × 250 nm) equipped with a precolumn (Knauer, Berlin, Germany) using an HPLC system consisting of two model LC-10 AD VP pumps, a SIL-10 AD VP injector, a SPD-10A VP UV–vis detector, a SCL-10A VP system controller, and CLASS-VP version 5.032 software (all from Shimadzu). The solvent system comprised 0.1% TFA in water (solvent A) and 0.08% TFA in 80% acetonitrile (solvent B), with a linear gradient from 10% to 70% of solvent B in 41 min with a flow rate of 1 mL/min. The peptide separation was monitored spectrophotometrically at 215 nm. Peptide-containing fractions were collected, dried in a Speed-Vac (Martin Christ, Osterode am Harz, Germany), resuspended in 30% methanol with 0.1% formic acid, and then analyzed using an HCTultra ETDII mass spectrometer (Bruker, Bremen, Germany) as previously described [40,41]. Peptide solutions were directly injected at a flow rate of 180 μL/h with a syringe pump (KD Scientific, Holliston, MA, USA) into an electrospray ionization ion source, operated in positive ion mode at a capillary voltage of 3.5 kV, a drying gas flow of 5 L/min, a nebulizer pressure of 10 psi, and an ion source temperature of 300 °C. The ion trap analyzer was set to MS mode, and the peptide identification was performed manually using DataAnalysis^TM^ 4.0 software (Bruker). All reagents of LC–MS purity were purchased from Sigma-Aldrich.

### 2.10. Statistical Analysis

To analyze the statistical significance, an unpaired *t*-test and one-way ANOVA test with Dunnett’s multiple comparisons were performed with GraphPad Prism software version 9.5.1 (GraphPad Software, La Jolla, CA, USA). Results on bar graphs or scatter plots are presented as mean ± standard deviation. The statistical significance levels were marked with * for *p* < 0.05, ** for *p* < 0.01, *** for *p* < 0.001, **** for *p* < 0.0001, and ns when not significant.

## 3. Results

### 3.1. Characteristics of C. albicans Biofilm-Derived EVs

**Figure 2:**
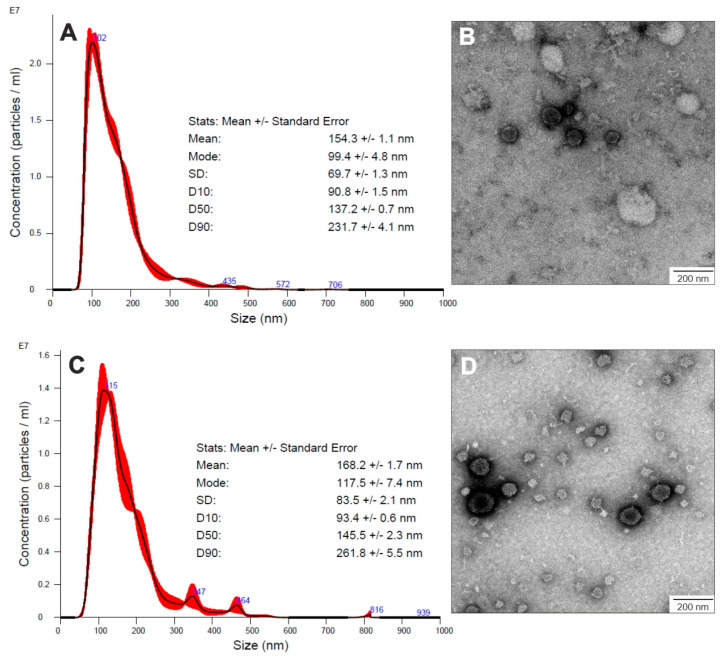
Characteristics of EVs produced by *C. albicans* biofilms. NTA particle size distribution analysis of *C. albicans* strain SC5314 (**A**) and strain 3147 (**C**); representative histograms of the average size distribution from three measurements of a single sample (black line) are presented. The presented numbers indicate the maxima of peaks, and the red areas indicate the standard deviation (SD) between measurements. The size parameters of the EVs are included. TEM images of EVs produced by *C. albicans* strain SC5314 (**B**) and strain 3147 (**D**). Scale bar: 200 nm.

Extracellular vesicles produced by two strains of *C. albicans*—SC5314 and 3147 (ATCC 10231)—were isolated with the differential centrifugation technique followed by ultracentrifugation from the culture medium collected after biofilm growth for 48 h, as described previously [19,20]. In both tested fungal strains, on the basis of TEM visualization (Figure 2), the presence of numerous spherical structures surrounded by a lipid bilayer was demonstrated in the obtained samples. In NTA analysis, a major distinct population of EVs with average sizes in the range of 90–260 nm could be distinguished (Figure 2). These observations were consistent with previous reports on the diameters and morphology of EVs produced by various *C. albicans* strains grown under diverse conditions and in different morphological forms [17,33], particularly considering results from studies on EVs from biofilm cells of *C. albicans* strain SN152 [20].

### 3.2. Increased Tolerance to Caspofungin

**Figure 3:**
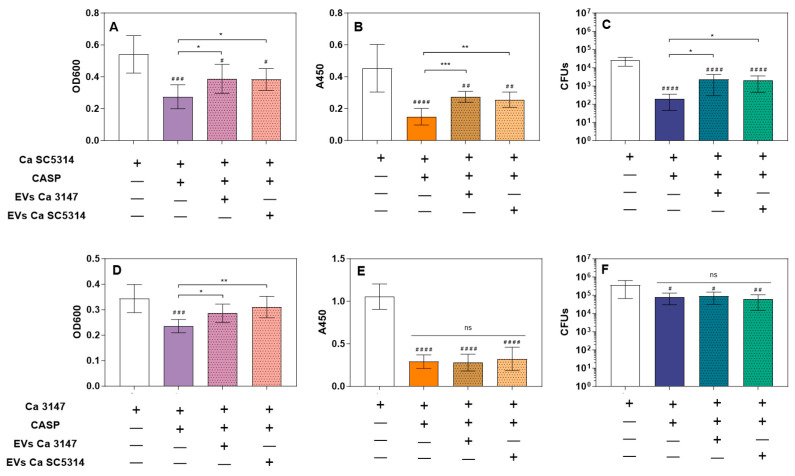
Formation of *C. albicans* biofilm produced by *C. albicans* strains SC5314 (**A**–**C**) and 3147 (**D**–**F**) in the presence of the antifungal drug caspofungin (CASP) and biofilm-derived EVs. The biofilm-forming capacity was examined by measuring optical density at 600 nm in the area scan mode (**A**,**D**), the metabolic activity of biofilm cells by the XTT reduction assay (**B**,**E**), and the viability of the biofilm-forming cells by CFU counting (**C**,**F**). A combined result of three independent experiments is presented. * *p* < 0.05, ** *p* < 0.01, *** *p* < 0.001, ns—not significant; when compared to biofilm without CASP ^#^ *p* < 0.05, ^##^ *p* < 0.01, ^###^ *p* < 0.001, ^####^ *p* < 0.0001.

For the verification of the effect of *C. albicans* biofilm-isolated EVs on resistance to caspofungin, EVs were added to *C. albicans* adhered cells at the first stage of biofilm formation, just after 90 min of germ tube initial adhesion. The incubation with EVs lasted 2 h and was followed by the introduction of caspofungin for the next 24 h. The final concentration of antifungal was 0.005 µg/mL, which still allowed for the observation of biofilm formation after initial cell adhesion. However, it also caused a noticeable reduction in biofilm thickness, the metabolic activity of biofilm cells, and their viability (the latter observation did not concern the *C. albicans* strain 3147) (Figure 3). It was demonstrated that vesicles released by mature 48-h biofilm formed by both tested *C. albicans* strains provided a protective effect for the developing biofilm structure of *C. albicans* strain SC5314 (Figure 3A–C). While for strain 3147, this was only observable for biofilm thickness measurements and there was no significant protective effect on the metabolic activity of biofilm cells (Figure 3D,E).

### 3.3. Biofilm Detachment

**Figure 4:**
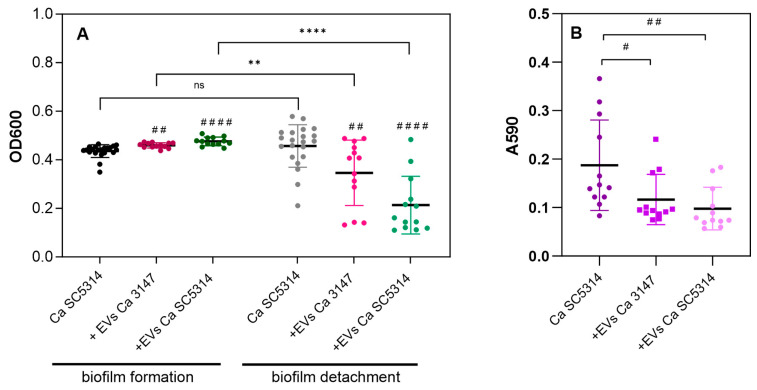
Formation of *C. albicans* SC5314 biofilm and its disruption in the presence of biofilm-derived EVs produced by *C. albicans* strains 3147 and SC5314. The biofilm thickness was investigated by measuring OD at 600 nm in the area scan mode (**A**) before the washing step (biofilm formation) and after washing (biofilm detachment), and by crystal violet staining (**B**). A combined result of four independent experiments is presented. The statistical significance levels compared to biofilm cultured without EVs were marked with ^#^ for *p* < 0.05, ^##^ for *p* < 0.01, ^####^ for *p* < 0.0001, and compared to the biofilm before the washing step with ** for *p* < 0.01, **** for *p* < 0.0001, and ns when not significant.

The analysis of the effect of EVs on the formation of biofilm by *C. albicans* strain SC5314 revealed a noticeable increase in the biofilm thickness after 24 h of growth with the addition of EVs isolated from mature biofilms formed by both tested strains of *C. albicans* (Figure 4A). However, after washing the surface-attached 24-h biofilm, the considerable detachment of biofilm fragments was repeatedly noticed for this strain. Biofilm disruption was observable macroscopically and microscopically and evidently visible as biofilm sections separated from the microplate surface. This phenomenon was occasionally observed for the control biofilm but was frequently reproducible in numerous experiments when the process of biofilm formation was accompanied by vesicles isolated from both strains, albeit more evident for the EVs of strain SC5314. This was directly perceptible after washing the biofilm, and spectrophotometric measurements of the optical density of the biofilm in the area scan mode, as well as staining the biofilm with crystal violet, confirmed these observations (Figure 4A,B; Supplementary Figure S1). Nevertheless, under the conditions used for strain 3147, no significant detachment of biofilm fragments was observed.

**Figure 5:**
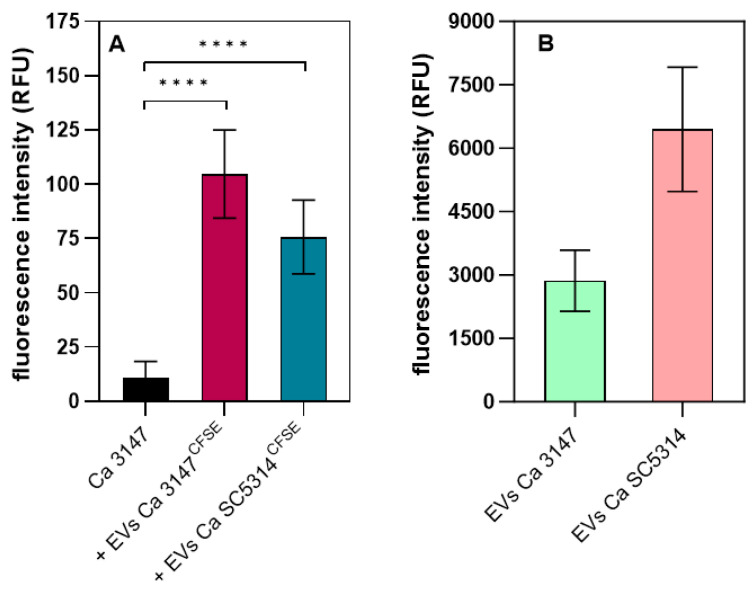
Localization of biofilm-derived EVs produced by *C. albicans* strains SC5314 and 3147 in *C. albicans* biofilm (**A**) and assessment of EV proteolytic activity (**B**). (**A**) A representative result from three biological replicates is presented. **** *p* < 0.0001 (**B**) The averaged results for four different EV batches obtained for each strain are shown.

With the use of fluorescently labeled EVs, we confirmed the location of vesicles and their contents within the biofilm being formed, as shown in Figure 5A for the *C. albicans* 3147 strain and for EVs from both species; for the SC5314 strain, such an analysis was very difficult due to the significant detachment of the biofilm after washing with buffer. Next, the proteolytic activity of *C. albicans* EVs was tested with the use of a casein derivative labeled with the green-fluorescent BODIPY^®^ FL dye (Figure 5B). The proteolytic activity demonstrated by EVs produced by both tested strains was revealed, and the reaction was carried out for 24 h at pH 7.4. That also corresponded to the conditions under which the fungal biofilm was formed in the presence of vesicles. Although this is not the optimal pH for the activity of *C. albicans* Saps, which are responsible for most of the proteolytic activity of *Candida* cells and were identified in EVs [20,27,37], the long reaction time and residual proteolytic activity also resulted in the hydrolysis of the substrate. This observation suggested the possibility of the participation of EVs and their proteolytic activity in disturbing biofilm structure.

**Figure 6:**
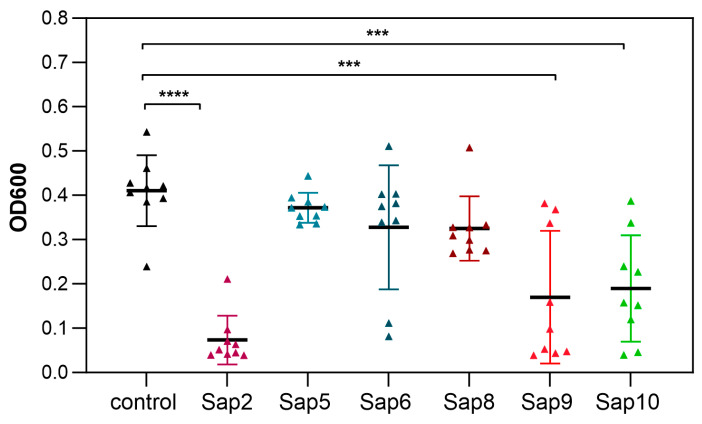
Formation of *C. albicans* SC5314 biofilm in the presence of *C. albicans* recombinant secreted aspartic proteinases (Saps). A combined result of three independent experiments is presented. Biofilm formed without the addition of recombinant Saps served as a control. *** *p* < 0.001 and **** *p* < 0.0001.

Therefore, the effect on biofilm stability was examined using selected recombinant Saps separately under the same conditions as those used for EVs during the process of biofilm formation. Proteomic analysis of the composition of EVs obtained and used in this study revealed the presence of several Saps in *C. albicans* vesicles (Supplementary Table S1), including Sap2, Sap5, Sap6, Sap8–10 for *C. albicans* strain SC5314, and Sap5, Sap6, and Sap9 for strain 3147. These particular Saps were therefore tested, and three of them were shown to be especially effective in biofilm detachment, including Sap2, Sap9, and Sap10 (Figure 6).

### 3.4. Degradation of NAT26

**Figure 7:**
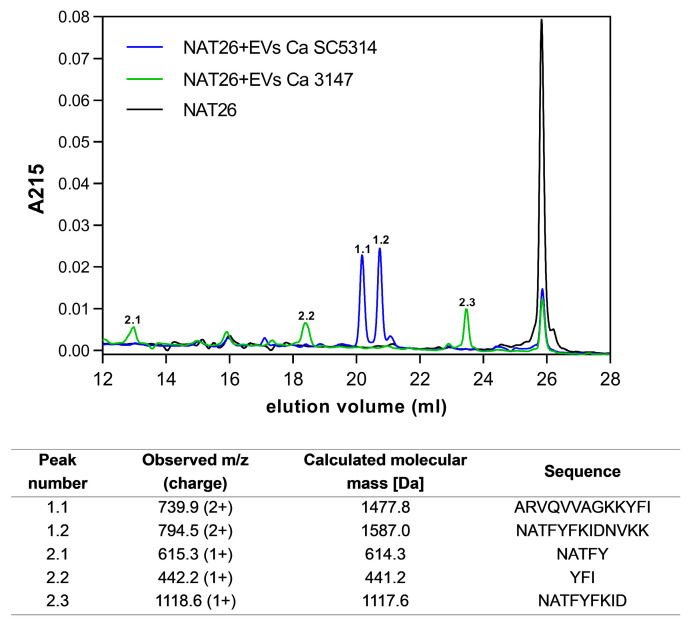
HPLC profiles of NAT26 peptide incubated with EVs produced by biofilms of *C. albicans* strain 3147 (green line) or strain SC5314 (blue line). In particular, fractions with annotated numbers and specified amino acid sequences were MS-identified and listed under the chromatograms.

Then, using HPLC separations of peptides, the degradation progress of the NAT26 antifungal peptide derived from human kininogen was performed after its incubation with EVs of both strains, maintaining a reaction time of 24 h and pH 7. The eluted fractions were collected, and then their molecular masses were determined by tandem mass spectrometry and compared with previously determined sequences of degradation products [40]. The degradation profile was different for EVs of both tested *C. albicans* strains, since for EVs from strain 3147, three major peaks were identified, whereas for EVs from strain SC5314, two distinct peaks were identified (Figure 7). The peptide fragments detected overlap with some peptides identified for the action of individual Saps on NAT26, as indicated in the previous work [40]. The shorter peptide fragments identified for EVs of strain 3147 comprise parts of a sequence of the longer NAT26 fragments identified for EVs of strain SC5314, which may indicate further proteolytic processing. Importantly, previous work [40] showed that even when these recombinant proteinases acted alone on NAT26, they significantly deprived it of its antifungal properties. Therefore, considering the possible synergistic effect of Saps in EVs, it may also result in a similar effect in the case of vesicular structures.

## 4. Discussion

Extracellular vesicles produced by *C. albicans* are richly equipped with various proteins and virulence factors, including numerous enzymes. Their activity may contribute to processes related to the physiology of fungi as well as the pathogenesis of infections they cause [20,42,43]. Molecules transported in vesicles may be more resistant to unfavorable environmental conditions and degradation [44] and act at a greater distance from pathogen cells settled in a particular host niche [4,6]. The involvement of *C. albicans* EVs in the formation and remodeling of the biofilm matrix, immunomodulatory properties, and resistance to the antifungal drug fluconazole have been repeatedly demonstrated [20,21,22,45,46]. Also, the presence of important virulence factors such as Saps in vesicles was confirmed, although their direct proteolytic activity, to the best of our knowledge, has not been previously reported on specific substrates.

Previous reports indicated that biofilm-derived EVs may contribute to a lower susceptibility of *C. albicans* biofilms to the antifungal drug fluconazole [20], and for *C. tropicalis* biofilms, such an effect of biofilm protection by EVs against the antifungals was also demonstrated for fluconazole and caspofungin [19]. In our previous work, when we added caspofungin to surface-attached *C. tropicalis* cells at the initial stage of biofilm formation, the biofilm thickness, cell metabolic activity, and viability were partially restored when EVs derived from mature, 48-h biofilms were introduced 90 min before the drug [19]. Caspofungin, an antifungal echinocandin, exhibits inhibitory action on β-1,3-glucan synthesis and causes disorders in cell wall structure and biofilm formation in *Candida*. Currently, resistance to caspofungin based on various mechanisms is also a demanding problem during candidal infections [47]. In this study, the results differed slightly depending on the *C. albicans* strain tested, but for both strains—3147 and SC5314—in the presence of cross-applied EVs with the subsequent introduction of caspofungin at subinhibitory concentration, the biofilm thickness increased considerably compared to biofilm without vesicles but with the applied antifungal drug. Additionally, for *C. albicans* strain SC5314, after caspofungin addition, the metabolic activity and viability of cells in biofilm increased in the presence of EVs. This may indicate an additional mechanism of reduced susceptibility to caspofungin by the vesicle-producing *C. albicans* biofilm, accompanying other previously described important resistance mechanisms [48]. Nevertheless, this may depend on the C. *albicans* strain and its specific susceptibility to the treatment, as for strain 3147 the effect was not so unequivocal.

Furthermore, in the case of *C. tropicalis*, the addition of EVs did not significantly affect the growth and stability of the biofilm (not influenced by antifungal drugs) [19]. However, in the case of analyses performed for *C. albicans* SC5314, the biofilm thickness was greater after the addition of EVs from both *C. albicans* strains. Additionally, after 24 h of growth, the biofilm state was different when the EVs from mature biofilms were constantly present. As described recently, *C. albicans* strain SC5314 is more efficient in filamentation and biofilm formation compared to 224 other *C. albicans* sequenced isolates. This is related to the heterozygosity of the locus of the gene *ROB1*, which encodes the zinc finger transcription factor that controls the process of biofilm formation [49,50]. This variation is based on a difference in one amino acid (*ROB1*^946S^ or *ROB1*^946P^ allele) but significantly affects the *C. albicans* phenotype, causing robust biofilm formation [50]. The second strain we tested, *C. albicans* 3147 (ATCC 10231), formed a thinner biofilm. The difference in biofilm detachment between strains was also significant because the thick biofilm formed by strain SC5314 was easily disturbed after rinsing, especially if its development was already accompanied by EVs from the initial stage. There was no such observation for strain 3147; the biofilm was stable in all tested conditions; however, EVs from this strain also had an impact on the detachment of the SC5314 biofilm, similar to EVs derived from the SC5314 strain. The added EVs of both strains were produced by mature biofilms and applied to initially adhered germ tubes. Then, their impact on the further stages of biofilm formation subsequent to the initiation of filamentation was monitored. It should be noted that the observed effects could directly depend on a specific molecular composition of these biofilm-derived EVs, particularly considering previous reports in which a significant impact of EVs on the filamentation process was detected and correlated with the origin of EVs [8,18]. A recent study by Honorato et al. demonstrated that the particular components (terpenes and fatty acids) of the EVs produced by *C. albicans* strain 90028 grown in the form of yeast cells were capable of inhibiting filamentation and biofilm formation [18]. However, somewhat different conclusions were presented in the study by Bitencourt et al. [8], when EVs were isolated from cultures of *C. albicans* strain ATCC 64548 grown in YPD medium at different pHs—6.3 or 7.4 as yeast or yeast-to-hyphal forms, respectively. In their study, these EVs were found to stimulate the formation of pseudohyphae, although specific vesicular molecules responsible for this phenomenon have not been reported [8]. Therefore, considering the complex composition of EVs influenced by their source and mechanisms of biogenesis, several different mechanisms involved in the control of biofilm initiation, formation, and dispersion could be hypothesized.

Biofilm detachment and biofilm cell dispersion are considered the final stages of biofilm formation, important for the further spreading of microbial cells from inhabited niches to other infectious sites and the initiation of the new biofilm, thus being an advantageous phenomenon for pathogens and being thoroughly studied [51]. The biofilm disaggregation and detachment phenomenon described for surface-attached biofilms is based on the release of large fragments of the microbial layer, most often under the influence of fluid shear and adhesion disturbance [52]. The occurrence of biofilm dispersion was previously assessed for *C. albicans* biofilm using methods similar to those used herein [53]. Furthermore, results presented in previous works specified the role in the dispersal of biofilm for certain proteins found in *C. albicans* EVs—i.e., Phr1, Sun41, and Pra1 proteins were proposed as inhibitors of biofilm dispersion [21]. Additionally, the important role of EV-contained quorum sensing molecules involved in the inhibitory regulation of filamentation and biofilm formation has been indicated [18]. Therefore, it can be concluded that the specific composition of EVs is key to regulating the biofilm, and an important mechanism for this may be the effect on filamentation [8,18]. In this work, we hypothesized some additional complementary effects of aspartyl proteinases found in EVs on the process of biofilm detachment by a mechanism associated with proteolysis in the biofilm cells’ environment. As *C. albicans* Sap proteinases are very abundant hydrolases in the biofilm-derived vesicles [20] and surface-exposed adhesive proteins play an important role in the attachment of biofilm cells to the inhabited surface [54,55], we decided to verify whether it is the proteolytic activity of EVs that contributes to the detachment of the biofilm. Undoubtedly, this is only one of the several possible mechanisms related to the dispersion of biofilm because several other vesicular molecules, not only proteins, may be involved in the processes related to biofilm regulation [18]. The proteolytic activity of the tested EV preparations obtained from both strains has been confirmed in this study using the fluorogenic proteinaceous substrate under conditions that were not optimal for Saps activity but favorable to the formation of a robust biofilm. Furthermore, the location of the vesicle contents in the formed biofilm has been demonstrated. Therefore, further analysis of the impact of individual Saps on biofilm stability was needed. We focused on these particular proteinases, whose presence in EV preparations obtained for the studies presented herein was confirmed by proteomics. In other published analyses, additional Saps were identified [20]. This could have been the consequence of the different types of LC–MS/MS equipment used as well as the use of distinct methods of sample preparation. Specifically, in our EV samples, we did not identify Sap4, while Sap5, Sap6, and Sap9 were identified in EVs from both investigated strains. Additionally, Sap2, Sap8, and Sap10 were identified in vesicles produced by *C. albicans* SC5314, as demonstrated herein and in other analyses [20].

The highest activity affecting biofilm stability was demonstrated by Sap2, Sap9, and Sap10. Sap2 is the major aspartic proteinase of broad substrate specificity and is secreted extracellularly by *C. albicans*, being involved in nutrient acquisition and associated with fungal virulence—host invasion, tissue damage, and immune evasion [27,56,57]. Although the optimum activity of Sap2 is at acidic pH, research presented by Lin et al. [57] showed that this proteinase might also be active at pH 7. Sap9 and Sap10, unlike other members of the family, are glycosylphosphatidylinositol-anchored N-glycosylated proteins and remain associated with the surface of fungal cells, performing various functions related to the cell wall integrity and processing of other surface proteins, as well as being involved in the interactions with the host [58,59]. Sap9 and Sap10 have a preference for hydrolysis of peptide bonds after dibasic (KR, KK) or monobasic (K, R) residues. Additionally, Sap10 cleaves bonds between F–S and H–N [58]. The optimal pH for the enzymatic activity of these two Saps is close to 7. Studies performed by Schild et al. [59] demonstrated that several cell wall proteins of *C. albicans*, including Cht2, Als2, Ywp1, Rhd3, Pga4, Ecm33, and Rbt5, were in vitro substrates for these proteinases [59], suggesting their important role in the regulation of adhesion and biofilm. Interestingly, biofilm-specific and abundant Sap5 and Sap6 [60] did not show frequent and repeated activity towards biofilm detachment under the conditions applied.

Considering the significant involvement of Saps in the proteolytic degradation of host proteins and peptides [27], a further issue to be verified was whether these enzymes located in EVs exhibit the ability to influence host molecules similarly to soluble or cell surface-anchored Saps. The verification of the host-directed proteolytic activity for biofilm-derived EVs was carried out using the NAT26 peptide with the sequence NATFYFKIDNVKKARVQVVAGKKYFI, comprising amino acid residues 294–319 in domain 3 of human kininogens [61]. NAT26 possesses potent antibacterial and anti-*Candida* properties [40,61], although *C. albicans* Saps can degrade this peptide and deprive it of its antifungal properties [40]. In the case of incubation of NAT26 with EVs from both *C. albicans* strains, degradation of the human peptide and the formation of fragments analogous to those observed in the case of the single Sap action were studied in more detail in a previous study [40]. The comparison of the degradation profiles for recombinant Saps and EVs indicates that in the case of EVs of strain 3147, Sap5, Sap6, and Sap9 may be involved in the degradation of NAT26, and in the case of strain SC5314—particularly Sap5 and Sap6. However, the participation of other Saps cannot be excluded. For strain 3147, the presence of all three proteinases was confirmed in the proteomic analyses in this study, as was the presence of Sap5 and Sap6 in the EVs of strain SC5314. Therefore, this observation corroborates the possible involvement of Sap proteinases located in fungal EVs in the degradation of host defense molecules in a similar way to soluble proteinases.

In the field of research on *C. albicans* EVs, many issues still remain difficult to solve, including the mechanisms of their biogenesis, cargo selection, secretion, and transit through the cell wall, content release, and fusion with target cells [62]. Moreover, specific functionalities of EVs related to the physiology of fungi, as well as their virulence, are constantly being discovered and documented. The rich and diverse vesicular content implies a number of potential functions of EVs, and the multi-component nature of these structures may be the reason for the multi-complexity of their interactions both with the microbial cells forming a complex biofilm community as well as during host-pathogen interactions. An in-depth exploration of these functions, especially in relation to the mechanisms of pathogenesis of fungal infections and resistance to antifungal drugs, may be a promising contribution to the proposal of more effective antifungal therapies.

## Data Availability

The datasets generated and analyzed during the current study are available in the Cracow Open Research Data Repository, https://doi.org/10.57903/UJ/ISYW8J (accessed on 2 September 2023). The mass spectrometry proteomics data have been deposited with the ProteomeXchange Consortium via the PRIDE partner repository with the dataset identifier PXD044271.

## Supplementary Materials

The following supporting information can be downloaded at: https://www.mdpi.com/article/10.3390/jof9111078/s1, Supplementary Table S1: Mass spectrometry identification of *Candida albicans* proteins in EVs. Supplementary Figure S1: Formation of *C. albicans* SC5314 biofilm and its detachment in the presence of biofilm-derived EVs produced by *C. albicans* strains 3147 and SC5314 (photograph of crystal violet staining).
