# Supplementary material for: Candida albicans Biofilm-Derived Extracellular Vesicles Are Involved in the Tolerance to Caspofungin, Biofilm Detachment, and Fungal Proteolytic Activity
Source: J Fungi (Basel). 2023 Nov 4;9(11):1078. doi: 10.3390/jof9111078 (PMC10672323; doi:10.3390/jof9111078)
Supplement: Supplementary file 1 [file jof-09-01078-s001.zip › Supplementary Figure S1.pdf]

Candida albicans biofilm-derived extracellular vesicles are involved in the tolerance to caspofungin, biofilm detachment and fungal proteolytic activity

Justyna Karkowska-Kuleta [1,*], Kamila Kulig[1], Grazyna Bras[1], Karolina Stelmaszczyk[1], Magdalena Surowiec[1,2], Andrzej Kozik[3], Elzbieta Karnas[4], Olga Barczyk-Woznicka[5], Ewa Zuba-Surma[4], Elzbieta Pyza[5] and Maria Rapala-Kozik[1]

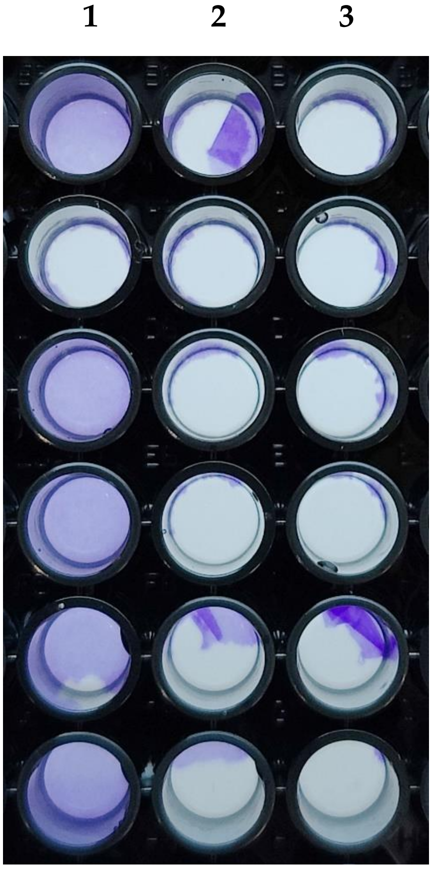

Supplementary Figure S1. Formation of *C. albicans* SC5314 biofilm (1) and its detachment in the presence of biofilm-derived EVs produced by *C. albicans* strain 3147 (2) and SC5314 (3). The biofilm thickness was investigated by crystal violet staining**.** A represenative result of experiment with six technical replicates is presented.
