# Supplementary material for: Candida albicans Biofilm-Derived Extracellular Vesicles Are Involved in the Tolerance to Caspofungin, Biofilm Detachment, and Fungal Proteolytic Activity
Source: J Fungi (Basel). 2023 Nov 4;9(11):1078. doi: 10.3390/jof9111078 (PMC10672323; doi:10.3390/jof9111078)
Supplement: Supplementary file 1 [file jof-09-01078-s001.zip › Supplementary Table S1.pdf]

Candida albicans biofilm-derived extracellular vesicles are involved in the tolerance to caspofungin, biofilm detachment and fungal proteolytic activity

Justyna Karkowska-Kuleta [1,*], Kamila Kulig[1], Grazyna Bras[1], Karolina Stelmaszczyk[1], Magdalena Surowiec[1,2], Andrzej Kozik[3], Elzbieta Karnas[4], Olga Barczyk-Woznicka[5], Ewa Zuba-Surma[4], Elzbieta Pyza[5] and Maria Rapala-Kozik[1]

Supplementary Table S1. Mass spectrometry identification of *Candida albicans* proteins in EVs. The proteomic analysis of extracellular vesicles was performed with samples prepared with a total amount of proteins equal to 20 µg. After digestion with Trypsin/Lys-C Mix the resulting peptides were analyzed using UltiMate 3000 RSLCnano System coupled with Q-Exactive mass spectrometer. The obtained lists of peaks were searched against the Swiss-Prot protein database with the locally installed MASCOT search engine using the taxonomy restrictions: Fungi. The Target Decoy PSM Validator was applied with the maximum false discovery rate (FDR) for peptides set to 0.01. Only proteins identified with a score >20 and a number of identified peptides ≥1 are included. MW – molecular weight; SC – sequence coverage; PSMs – peptide sequence matches; AAs – amino acids.

Accession	Protein description	Gene	MW [kDa]	Calc. pI	Score	SC (%)	# Proteins	# Unique Peptides	# Peptides	# PSMs	# AAs
				Candida albicans **3147 (ATCC 10231)**							
P43094	Candidapepsin-5	SAP5	45.6	6.38	2643.08	62.92	1	21	21	62	418
Q92211	Glyceraldehyde-3-phosphate dehydrogenase	TDH1	35.8	7.12	1864.36	77.01	20	22	22	39	335
Q59NP5	Secreted beta-glucosidase SUN41	SUN41	43.7	4.84	1806.00	35.17	1	10	10	38	418
Q59XX2	Cell surface mannoprotein MP65	MP65	39.2	5.45	1756.19	46.56	1	13	13	34	378
O74623	Agglutinin-like protein 3	ALS3	119.9	4.58	1662.84	14.57	4	10	11	31	1119
P41797	Heat shock protein SSA1	SSA1	70.3	5.17	1450.04	51.07	12	9	26	38	656
P43076	pH-responsive protein 1	PHR1	59.4	5.07	1383.55	47.63	3	23	23	37	548
Q5AL03	Hyphally regulated cell wall protein 1	HYR1	91.9	4.65	1355.62	18.93	2	16	16	41	919
P87020	pH-regulated antigen PRA1	PRA1	33.1	5.25	1329.70	45.82	1	11	11	38	299
P46598	Heat shock protein 90 homolog	HSP90	80.8	4.88	1320.64	45.12	8	30	30	40	707
Q5AF03	Glyoxalase 3	GLX3	25.8	4.83	1276.94	67.80	1	16	16	32	236
O13287	6-phosphogluconate dehydrogenase, decarboxylating	DOR14	56.9	6.57	1241.23	61.70	5	25	25	34	517
P30575	Enolase 1	ENO1	47.2	5.81	1161.07	56.14	12	19	19	24	440
P83779	Pyruvate decarboxylase	PDC11	62.4	5.58	1108.35	48.32	2	16	16	24	567
P28877	Plasma membrane ATPase 1	PMA1	97.4	4.96	1080.76	30.95	7	14	20	25	895
P46587	Heat shock protein SSA2	SSA2	70.0	5.06	1064.77	33.95	4	3	18	26	645
Q9URB4	Fructose-bisphosphate aldolase	FBA1	39.2	6.06	1011.24	64.35	3	17	17	25	359
Q59PT0	V-type proton ATPase subunit B	VMA2	57.2	5.03	884.59	56.45	5	2	22	27	512
C4YJQ8	Elongation factor 2	EFT2	93.3	6.47	873.95	39.55	13	27	27	31	842
P46273	Phosphoglycerate kinase	PGK1	45.2	6.48	805.23	56.59	20	15	21	29	417
P43095	Candidapepsin-6	SAP6	45.4	8.21	766.64	41.63	4	13	13	20	418
C4YG73	Vacuolar protein sorting/targeting protein 10	VPS10	179.2	5.03	749.37	17.15	1	11	22	26	1586
Q5AB48	Secreted protein RBT4	RBT4	37.4	4.55	724.59	32.68	1	6	6	14	358
Q9P8W1	Lipase 4	LIP4	49.5	6.18	716.76	38.13	3	16	16	21	459
O94039	Transketolase 1	TKT1	73.7	5.82	669.14	45.05	2	22	22	25	677
P14235	Actin	ACT1	41.7	5.69	639.96	49.47	26	9	15	23	376

Q5AIR7	Glucan endo-1,3-beta-D-glucosidase 1	ENG1	124.0	5.40	635.68	15.81	2	14	14	15	1145
P87206	ATP-dependent RNA helicase eIF4A	TIF1	44.6	5.36	625.69	57.93	39	19	20	23	397
P83780	Glucose-6-phosphate isomerase	PGI1	61.1	6.40	603.53	42.73	7	18	18	23	550
P43070	Glucan 1,3-beta-glucosidase	BGL2	33.5	4.78	602.79	44.48	2	9	9	16	308
Q59TP1	Cell wall protein RBT1	RBT1	73.6	4.34	569.48	14.56	1	7	7	9	721
Q5AJB1	V-type proton ATPase catalytic subunit A	TFP1	67.6	5.27	545.35	45.06	7	19	19	19	617
C4YKT4	Ras-like protein 1	RAS1	32.2	4.67	531.33	39.24	12	7	7	9	288
Q5AD07	Cell surface Cu-only superoxide dismutase 5	SOD5	23.6	6.87	529.88	33.33	1	6	6	16	228
P22011	Peptidyl-prolyl cis-trans isomerase	CYP1	17.6	7.97	529.44	34.57	3	8	8	11	162
P83776	Hexokinase-2	HXK2	53.4	5.55	525.04	41.74	3	12	13	15	484
P87222	Ribosome-associated molecular chaperone SSB1	SSB1	66.4	5.38	516.26	33.28	12	15	15	15	613
P40953	Chitinase 2	CHT2	60.8	4.89	498.60	21.44	1	8	8	11	583
Q5AFA2	Extracellular glycosidase CRH11	CRH11	46.7	4.96	483.12	22.52	1	13	13	18	453
Q5A8T4	Agglutinin-like protein 1	ALS1	132.8	4.45	469.96	9.13	2	6	7	11	1260
Q9P940	Triosephosphate isomerase	TPI1	26.6	6.01	459.52	57.66	1	10	10	12	248
P43098	Fatty acid synthase subunit alpha	FAS2	207.5	5.62	454.82	10.24	6	15	15	15	1885
P29717	Glucan 1,3-beta-glucosidase	XOG1	50.0	5.64	453.41	50.68	3	13	13	17	438
P34731	Fatty acid synthase subunit beta	FAS1	227.8	6.02	449.67	11.34	2	17	17	17	2037
P0CY35	Elongation factor 1-alpha 1	TEF1	50.0	9.03	444.53	30.13	25	11	11	17	458
Q8NJN3	Acetyl-coenzyme A synthetase 2	ACS2	73.8	6.13	426.50	21.61	13	13	15	15	671
O94049	Acetyl-coenzyme A synthetase 1	ACS1	75.1	6.32	414.99	27.26	6	15	17	17	675
P83775	Putative NADPH-dependent methylglyoxal reductase GRP2	GRP2	37.6	6.44	413.63	37.54	1	9	9	9	341
P53696	Profilin	PFY1	13.8	5.45	398.33	60.32	1	4	4	7	126
Q5AKU5	Secreted beta-glucosidase SIM1	SIM1	39.4	4.87	396.05	23.66	1	6	6	8	372
P83781	Mitochondrial outer membrane protein porin	POR1	29.7	8.57	395.66	39.36	1	9	9	11	282
O42766	14-3-3 protein homolog	BMH1	29.5	4.81	378.99	48.86	8	12	12	14	264
Q00310	Glycolipid 2-alpha-mannosyltransferase 1	MNT1	50.0	6.90	336.30	41.07	1	12	13	13	431
Q59MN0	Vacuolar protein 8	VAC8	63.4	5.07	328.48	14.70	5	7	7	8	585
O94072	V-type proton ATPase subunit E	VMA4	25.4	5.40	326.45	41.59	1	8	8	9	226
P46614	Pyruvate kinase	CDC19	55.4	6.99	316.95	25.40	12	9	9	10	504
Q5AAU5	Cell wall acid trehalase ATC1	ATC1	120.2	5.21	305.41	10.58	1	8	8	9	1078
P22274	ADP-ribosylation factor	ARF1	20.2	5.40	303.25	45.25	1	2	5	7	179
P82612	Phosphoglycerate mutase	GPM1	27.4	6.16	290.83	42.34	1	8	8	9	248
Q59M70	NADH-cytochrome b5 reductase 2	MCR1	33.4	8.48	280.24	33.22	2	9	9	10	301
O13359	Kexin	KEX2	105.1	5.03	279.11	7.57	1	8	8	11	938

P10875	Tubulin beta chain	TUB2	49.9	4.74	275.68	19.82	9	6	6	6	449
P83784	Heat shock protein SSC1, mitochondrial	SSC1	69.7	5.60	249.87	8.95	3	4	4	7	648
P53698	Cytochrome c	CYC1	12.2	9.66	246.89	32.73	4	4	4	7	110
P31225	Corticosteroid-binding protein	CBP1	55.5	5.29	243.62	25.15	1	10	10	10	489
A0A1D8PH78	Farnesyl pyrophosphate synthase	ERG20	40.7	4.98	234.97	20.51	1	5	5	6	351
P43071	Multidrug resistance protein CDR1	CDR1	169.8	6.98	232.77	7.79	7	9	9	9	1501
P10977	Vacuolar aspartic protease	APR1	45.4	4.83	232.42	26.01	1	5	5	5	419
Q5AJY5	1,3-beta-glucanosyltransferase PGA4	PGA4	49.0	4.74	230.29	16.63	1	5	5	6	451
P0CU34	Peroxiredoxin TSA1-B	TSA1B	21.8	5.06	229.49	47.96	4	7	7	7	196
Q59SU1	Candidapepsin-9	SAP9	58.4	5.25	222.75	10.48	2	6	6	6	544
A0A1D8PCX8	Large ribosomal subunit protein eL6	RPL6	19.8	10.21	219.30	34.09	3	5	5	5	176
Q5A302	Endoplasmic reticulum vesicle protein 25	ERV25	24.5	7.12	217.72	21.40	1	4	4	5	215
O74226	Cell wall synthesis protein KRE9	KRE9	29.1	8.18	209.37	19.19	1	3	3	3	271
P83774	Small ribosomal subunit protein RACK1	ASC1	34.5	6.54	197.20	20.19	1	5	5	5	317
P0CY31	Ras-related protein SEC4	SEC4	23.1	5.47	196.55	37.14	1	6	6	6	210
P46592	Glycolipid 2-alpha-mannosyltransferase 2	MNT2	54.5	6.67	195.38	24.73	1	10	11	11	461
P87066	Tubulin alpha chain	TUB1	49.9	5.06	192.80	30.36	16	10	10	10	448
Q5ACR4	Protein OS-9 homolog	YOS9	29.4	5.87	192.47	23.64	1	5	5	5	258
P43077	Beta-hexosaminidase	HEX1	63.5	5.59	187.87	9.61	1	4	4	4	562
Q5A2J7	Calcium channel YVC1	YVC1	77.3	5.08	187.40	8.74	1	5	5	5	675
P83783	Adenosylhomocysteinase	SAH1	49.0	5.59	185.36	22.67	4	9	9	9	450
O42825	GTP-binding protein RHO1	RHO1	22.0	5.73	183.09	42.93	7	6	6	7	198
O93827	Mannose-1-phosphate guanyltransferase	MPG1	40.0	6.30	179.74	29.83	9	8	8	8	362
Q5ABU8	Beta-mannosyltransferase 6	BMT6	74.5	7.72	177.44	15.17	1	8	8	8	646
P28870	FK506-binding protein 1	RBP1	13.3	7.18	176.79	33.06	1	3	3	6	124
P83777	Inorganic pyrophosphatase	IPP1	32.1	5.26	176.78	29.51	9	6	6	6	288
Q5ABP8	Protein ROT1	ROT1	29.9	7.80	176.60	21.54	2	5	5	5	260
Q5A8I8	Induced during hyphae development protein 1	IHD1	36.9	4.34	172.87	8.42	1	2	2	3	392
C4YS59	Vacuolar membrane protease	CAWG_04918	93.7	5.29	172.57	7.77	7	5	5	6	837
Q59NP1	Copper transport protein CTR1	CTR1	27.8	6.93	170.86	21.91	1	4	4	4	251
P87220	V-type proton ATPase subunit D	VMA8	30.0	5.85	170.67	22.85	3	5	5	6	267
P31353	Phosphomannomutase	PMM1	29.0	5.69	163.51	16.67	2	3	3	3	252
A0A1D8PSV5	Small ribosomal subunit protein uS3	RPS3	27.3	9.23	160.22	19.92	2	4	4	4	251
P40954	Chitinase 3	CHT3	60.0	4.91	157.69	11.99	1	4	4	6	567
Q5AJF7	Large ribosomal subunit protein uL11	RPL12	17.8	9.51	153.74	30.91	4	4	4	4	165

O94083	Eukaryotic translation initiation factor 5A	ANB1	17.1	5.05	153.52	37.34	3	4	4	5	158
A0A1D8PCI6	Small ribosomal subunit protein eS4	RPS42	29.4	10.15	143.66	20.99	3	5	5	6	262
Q96VB9	Heat shock protein homolog SSE1	MSI3	78.5	5.30	139.99	10.41	5	6	6	6	701
P43067	Alcohol dehydrogenase 1	ADH1	36.9	6.44	138.33	17.43	4	6	6	6	350
Q59WF4	Alpha-1,2-mannosyltransferase MNN2	MNN2	69.1	6.27	138.01	9.21	1	5	5	5	597
A0A1D8PQQ5	Small ribosomal subunit protein uS13	RPS18	17.0	10.35	136.33	22.07	2	3	3	3	145
Q5AP66	Phosphatidylinositol transfer protein SFH5	SFH5	36.6	5.33	134.62	14.06	1	4	4	4	320
A0A1D8PH52	Acetyl-CoA acetyltransferase	ERG10	41.9	6.90	134.33	9.70	1	3	3	3	402
Q5AEN1	Cytochrome c peroxidase, mitochondrial	CCP1	40.7	6.34	124.83	12.84	1	4	4	4	366
P83778	Malate dehydrogenase, cytoplasmic	MDH1	36.0	5.62	124.51	17.51	1	3	3	3	337
A0A1D8PFG4	Large ribosomal subunit protein eL27	RPL27A	15.5	10.18	122.77	30.15	2	4	4	4	136
Q5AHH4	Small heat shock protein 21	HSP21	21.5	5.35	119.69	15.34	1	3	3	4	189
Q5A762	Multiple drug resistance-associated protein-like transporter 1	MLT1	180.6	6.32	118.82	3.86	2	4	4	4	1606
Q5ABA2	Ceramide-binding protein SVF1	SVF1	43.0	5.07	117.88	8.64	1	3	3	3	382
A0A1D8PK43	Large ribosomal subunit protein eL18	RPL18	20.8	11.80	116.31	22.58	2	4	4	4	186
C4YFX2	Transcriptional repressor TUP1	TUP1	57.5	6.00	108.77	10.37	2	4	4	4	511
Q5A860	Translationally-controlled tumor protein homolog	TMA19	18.5	4.46	107.31	16.77	2	2	2	2	167
P25997	Elongation factor 3	CEF3	116.9	5.73	105.37	4.95	5	4	4	5	1050
A0A1D8PEY9	Small ribosomal subunit protein eS17	RPS17B	15.7	10.46	104.37	8.03	1	1	1	1	137
A0A1D8PCG7	Small ribosomal subunit protein eS21	RPS21B	9.6	8.15	104.20	28.74	2	2	2	2	87
A0A1D8PDT3	Small ribosomal subunit protein uS11	RPS14B	14.0	10.90	104.06	23.48	5	2	2	2	132
O74660	Agglutinin-like protein 4 (Fragments)	ALS4	90.1	4.60	102.92	1.71	2	1	1	1	875
Q59S78	Small COPII coat GTPase SAR1	SAR1	21.5	5.59	100.12	23.16	5	3	3	3	190
A0A1D8PTW6	Hydroxymethylglutaryl-CoA synthase	ERG13	49.7	5.97	99.95	5.76	1	2	2	2	451
P30574	Carboxypeptidase Y	CPY1	61.0	5.47	97.64	6.27	1	3	3	3	542
Q5A0X8	Secreted hemophore CSA2	CSA2	15.1	7.50	96.56	32.65	1	3	3	3	147
A0A1D8PPE0	Small ribosomal subunit protein uS15	RPS13	16.9	10.23	96.51	20.53	5	4	4	4	151
Q5AFN8	Covalently-linked cell wall protein 14	SSR1	22.5	4.68	96.42	5.98	1	1	1	2	234
Q5AG43	Small ribosomal subunit protein uS7]	RPS5	25.3	8.70	95.93	11.56	1	2	2	2	225
Q5AJC0	Extracellular glycosidase UTR2	UTR2	51.7	4.73	95.85	12.13	1	4	4	4	470
A0A1D8PK40	Large ribosomal subunit protein eL19	RPL19A	21.9	11.30	95.59	10.53	1	2	2	2	190
P39826	Cell division control protein 3	CDC3	47.8	6.64	95.14	6.97	3	3	3	3	416
A0A1D8PLC9	Large ribosomal subunit protein eL20	RPL20B	20.3	10.24	94.23	20.35	1	3	3	3	172
P0CY19	Deoxyuridine 5'-triphosphate nucleotidohydrolase	DUT1	16.9	5.92	93.72	21.38	2	2	2	2	159
Q59Y31	Yeast-form wall Protein 1	YWP1	54.2	4.81	93.64	4.50	1	2	2	4	533

Q5A4M8	Protein SUR7	SUR7	29.9	7.69	93.13	14.07	1	3	3	3	270
A0A1D8PK61	Small ribosomal subunit protein eS19	RPS19A	16.1	9.42	88.97	13.10	1	3	3	3	145
A0A1D8PTR4	Small ribosomal subunit protein uS14	CAALFM_CR08480CA	6.6	9.76	88.88	44.64	2	3	3	4	56
O13401	Superoxide dismutase [Mn], mitochondrial	SOD2	26.2	8.73	88.77	13.68	1	2	2	2	234
P83782	Cytochrome b-c1 complex subunit 2, mitochondrial	QCR2	39.5	5.57	87.02	5.35	1	2	2	2	374
Q5AEN2	Large ribosomal subunit protein uL6	RPL9B	21.7	9.51	86.47	14.14	1	2	2	2	191
Q59LS1	Large ribosomal subunit protein uL3	RPL3	43.9	10.26	86.13	4.63	6	2	2	3	389
Q5A5S7	Autophagy-related protein 27	ATG27	28.3	5.53	84.81	9.92	1	2	2	2	252
A0A1D8PFL9	Large ribosomal subunit protein eL14	RPL14	14.7	10.90	84.21	24.43	1	3	3	3	131
A0A1D8PQN0	Small ribosomal subunit protein eS28	RPS28B	7.5	10.36	83.29	31.34	1	2	2	2	67
Q59YF0	Protein transport protein SSO2	SSO2	34.3	5.36	82.04	9.15	1	2	2	2	295
P82610	5-methyltetrahydropteroyltriglutamate--homocysteine methyltransferase	MET6	85.6	5.60	81.20	5.61	2	3	3	3	767
A0A1D8PM41	Large ribosomal subunit protein eL22	RPL22B	14.1	5.36	80.61	23.39	1	2	2	2	124
Q5A7K0	Small ribosomal subunit protein eS24	RPS24	15.5	10.87	80.49	17.78	2	2	2	2	135
Q5A389	Small ribosomal subunit protein uS10	RPS20	13.3	9.94	79.19	22.69	2	3	3	3	119
Q59KI0	UTP--glucose-1-phosphate uridylyltransferase	UGP1	55.5	6.73	78.72	8.60	1	3	3	3	500
A0A1D8PF08	Large ribosomal subunit protein uL2	RPL2	27.3	10.71	77.80	22.05	4	4	4	4	254
Q5ANE3	Non-classical export protein 102	NCE102	18.1	9.01	76.84	20.59	1	2	2	2	170
A0A1D8PHW1	Large ribosomal subunit protein uL5	RPL11	19.8	9.95	76.70	8.05	4	1	1	1	174
O13426	Serine hydroxymethyltransferase, cytosolic	SHM2	52.0	7.20	74.67	3.62	4	2	2	2	470
Q59R28	Alpha-1,2-mannosyltransferase MNN26	MNN26	87.2	6.52	73.39	5.03	1	4	4	4	756
P43102	Ubiquitin-conjugating enzyme E2 4	UBC4	16.3	7.40	71.37	12.24	6	2	2	2	147
Q5A940	Multiprotein-bridging factor 1	MBF1	16.3	10.07	71.19	17.88	2	4	4	4	151
A0A1D8PGY8	Small ribosomal subunit protein uS4	RPS9B	21.7	10.20	67.74	16.93	6	3	3	3	189
A0A1D8PC43	Diphosphomevalonate decarboxylase	MVD	39.5	6.46	66.30	6.91	1	2	2	2	362
Q5AD72	Alpha-1,2-mannosyltransferase MNN24	MNN24	83.0	5.96	65.31	2.96	1	2	2	2	709
O59931	Large ribosomal subunit protein eL13	RPL13	23.0	10.61	65.17	13.86	1	3	3	4	202
P43065	Saccharopine dehydrogenase [NAD(+), L-lysine-forming]	LYS1	42.4	5.44	64.75	7.85	1	3	3	3	382
A0A1D8PHH4	Large ribosomal subunit protein eL33	RPL33A	12.1	10.78	60.30	17.76	1	1	1	1	107
A0A1D8PPN6	Large ribosomal subunit protein eL32	RPL32	14.9	10.54	59.67	16.03	1	2	2	2	131
Q5AQ76	Protein transport protein SEC24	SEC24	102.0	5.40	59.34	3.13	1	2	2	2	928
Q5AIB8	Large ribosomal subunit protein uL16	RPL10	25.2	10.08	56.98	13.18	1	1	1	1	220
Q8TGH6	Guanosine-diphosphatase	GDA1	65.9	5.94	56.59	4.67	1	3	3	3	599
Q5APQ8	Putative alpha-1,3-mannosyltransferase MNN12	MNN12	97.5	8.16	56.33	1.09	1	1	1	1	828
Q9P8V9	Lipase 8	LIP8	49.7	5.49	55.29	2.17	1	1	1	1	460

A0A1D8PFV1	Large ribosomal subunit protein uL4	RPL4B	39.2	10.74	53.92	5.23	1	1	1	1	363
O13289	Peroxisomal catalase	CAT1	54.8	6.65	52.97	5.98	1	2	2	2	485
Q9UVL1	Non-histone chromosomal protein 6	NHP6	10.5	9.70	52.83	14.13	1	1	1	1	92
Q5APC0	Golgi apparatus membrane protein TVP18	TVP18	18.8	7.74	51.75	13.87	1	1	1	1	173
Q9P926	Nuclear transport factor 2	NTF2	14.2	4.63	50.97	12.90	5	2	2	2	124
Q5A846	Beta-mannosyltransferase 3	BMT3	62.9	6.89	50.72	7.47	1	3	3	3	549
P46250	SEC14 cytosolic factor	SEC14	34.7	6.40	50.03	2.99	1	1	1	1	301
A0A1D8PCW6	Small ribosomal subunit protein uS9	RPS16A	15.7	10.29	48.55	14.08	6	2	2	2	142
Q5A6A1	Large ribosomal subunit protein eL24	RPL24A	17.4	11.37	48.45	7.10	3	1	1	1	155
Q5A6R1	Large ribosomal subunit protein eL15	RPL15A	24.3	11.34	48.03	12.75	3	3	3	3	204
Q5AJ93	Small ribosomal subunit protein eS7	RPS7A	21.2	9.92	47.83	7.53	1	1	1	1	186
A0A1D8PDL6	60S ribosomal protein L7-A	RPL7A	27.4	10.24	47.23	15.77	1	2	2	2	241
Q9UVJ4	Large ribosomal subunit protein uL1	RPL10A	24.4	9.76	47.15	9.68	4	2	2	2	217
Q5AI15	Polyadenylate-binding protein, cytoplasmic and nuclear	PAB1	70.4	5.29	46.80	3.18	1	2	2	2	629
A0A1D8PNQ6	Small ribosomal subunit protein eS25	RPS25B	11.6	10.15	46.40	9.52	5	1	1	1	105
A0A1D8PGY0	Large ribosomal subunit protein eL21	RPL21A	18.0	10.33	46.11	11.25	3	2	2	2	160
A0A1D8PHF5	Large ribosomal subunit protein eL31	RPL31B	13.0	9.82	46.08	7.14	1	1	1	1	112
P87024	Beta-glucan synthesis-associated protein SKN1	SKN1	83.7	5.15	45.92	2.44	1	1	1	1	737
Q5AKA5	Cys-Gly metallodipeptidase DUG1	DUG1	53.6	5.24	45.46	2.89	1	1	1	1	485
Q5A748	Sorting nexin-3	SNX3	18.4	9.44	45.46	5.10	1	1	1	1	157
A0A1D8PEL1	Mevalonate kinase	ERG12	47.0	5.68	45.10	2.55	1	1	1	1	431
Q5AGD1	Acyl-protein thioesterase 1	CAALFM_C502400WA	25.3	6.39	44.92	6.49	1	1	1	1	231
P0CY33	Cell division control protein 42 homolog	CDC42	21.2	6.54	44.88	5.76	1	1	1	1	191
Q59UT4	GPI-anchored hemophore RBT5	RBT5	24.2	4.37	43.78	4.15	2	1	1	1	241
Q5A900	Small ribosomal subunit protein uS5	RPS21	26.9	10.24	43.51	8.84	1	2	2	2	249
A0A1D8PL99	Small ribosomal subunit protein eS6	RPS6A	27.1	10.15	43.42	5.08	2	1	1	1	236
Q5ANA8	Metacaspase-1	MCA1	49.2	5.69	41.71	2.68	1	1	1	1	448
A0A1D8PI15	Small ribosomal subunit protein eS10A	RPS10	13.8	9.83	41.56	11.86	1	1	1	1	118
O94200	ATP-dependent 6-phosphofructokinase subunit beta	PFK2	104.0	6.35	40.70	0.95	1	1	1	1	946
Q59TE0	Large ribosomal subunit protein uL22	RPL17B	21.0	10.77	40.11	5.41	1	1	1	1	185
P52498	Ras-related protein RSR1	RSR1	27.6	5.21	39.46	4.44	1	1	1	1	248
P53704	Glutamine--fructose-6-phosphate aminotransferase [isomerizing]	GFA1	79.2	6.24	38.40	3.09	1	1	1	1	713
Q59WG0	Adenosine 5'-monophosphoramidase HNT1	HNT1	17.0	6.86	38.28	11.18	1	1	1	1	152
P53707	37 kDa cell surface protein	CSP37	37.0	7.06	37.51	3.12	1	1	1	1	321
Q5A477	GDP-mannose transporter	VRG4	41.2	9.36	35.08	2.70	1	1	1	1	371

A0A1D8PK22	Small ribosomal subunit protein uS19	RPS15	15.9	10.32	34.86	17.61	1	2	2	2	142
Q59T44	Small ribosomal subunit protein eS8	RPS8A	22.7	11.09	34.02	7.28	1	1	1	1	206
A0A1D8PTI7	Small ribosomal subunit protein eS27	RPS27	9.0	8.73	33.34	19.51	1	1	1	1	82
Q5ANL6	13 kDa ribonucleoprotein-associated protein	SNU13	13.6	7.97	33.02	19.05	2	1	1	1	126
A0A1D8PH21	Large ribosomal subunit protein eL36	RPL36	11.1	11.40	32.87	10.10	2	1	1	1	99
O74261	Heat shock protein 60, mitochondrial	HSP60	60.1	5.30	31.75	2.12	1	1	1	1	566
Q5AIA1	Glucan 1,3-beta-glucosidase 2	EXG2	54.5	5.53	31.40	4.18	1	2	2	2	479
O94201	ATP-dependent 6-phosphofructokinase subunit alpha	PFK1	108.5	6.62	31.18	0.81	1	1	1	1	987
P52495	Ubiquitin-activating enzyme E1 1	UBA1	114.2	5.01	30.72	1.96	1	1	1	1	1021
Q9UVX1	Lysophospholipase 3	PLB3	81.4	4.84	30.33	1.06	1	1	1	1	754
P34948	Mannose-6-phosphate isomerase	PMI1	48.8	5.33	29.96	1.59	1	1	1	1	441
Q92410	Alpha,alpha-trehalose-phosphate synthase [UDP-forming]	TPS1	54.4	6.23	29.38	5.86	12	2	2	2	478
Q12572	L-2-aminoadipate reductase large subunit	LYS2	154.6	6.87	28.74	0.72	1	1	1	1	1391
Q5AB87	Large ribosomal subunit protein uL13	RPL16A	22.6	10.32	28.41	4.00	1	1	1	1	200
P82611	Aconitate hydratase, mitochondrial	ACO1	84.2	6.39	26.89	1.29	1	1	1	1	777
Q5AP65	Protein FMP52, mitochondrial	FMP52	24.3	8.98	26.42	3.49	1	1	1	1	229
P43060	Phosphoribosylaminoimidazole-succinocarboxamide synthase	ADE1	32.9	5.50	25.16	7.22	1	2	2	2	291
P34725	Phospho-2-dehydro-3-deoxyheptonate aldolase, phenylalanine-inhibited	ARO3	40.7	7.11	24.91	1.90	5	1	1	1	368
Q5A893	F-actin-capping protein subunit alpha	CAP01	32.0	5.52	24.83	5.71	1	1	1	1	280
Q8J0Q0	Mannosyl-oligosaccharide 1,2-alpha-mannosidase	MNS1	64.6	5.11	24.35	1.77	1	1	1	1	565
P83773	Acetyl-CoA hydrolase	ACH1	58.0	6.92	22.74	4.20	1	2	2	2	524
Q9HEW1	cAMP-dependent protein kinase regulatory subunit	BCY1	50.3	5.48	21.06	2.40	1	1	1	1	459

Accession	Protein description		MW [kDa]	Calc. pI	Score	SC (%)	# Proteins	# Unique Peptides	# Peptides	PSMs	# AAs
					***Candida albicans* SC5314**						
P43094	Candidapepsin-5	SAP5	45.6	6.38	4198.47	65.31	1	26	26	118	418
P41797	Heat shock protein SSA1	SSA1	70.3	5.17	1844.48	54.42	13	12	30	50	656
P46598	Heat shock protein 90 homolog	HSP90	80.8	4.88	1760.70	49.93	7	35	35	50	707
Q5AC08	Candidapepsin-6	SAP6	45.4	8.03	1678.97	70.10	3	1	25	51	418
P46587	Heat shock protein SSA2	SSA2	70.0	5.06	1639.18	48.37	3	7	24	41	645
P43076	pH-responsive protein 1	PHR1	59.4	5.07	1594.82	47.63	3	24	24	41	548
O74623	Agglutinin-like protein 3	ALS3	119.9	4.58	1571.55	25.02	4	14	15	33	1119
Q92211	Glyceraldehyde-3-phosphate dehydrogenase	TDH1	35.8	7.12	1542.72	77.01	16	18	22	38	335
P30575	Enolase 1	ENO1	47.2	5.81	1428.33	60.23	13	20	20	29	440

P87020	pH-regulated antigen PRA1	PRA1	33.1	5.25	1357.16	45.82	1	12	12	46	299
Q59NP5	Secreted beta-glucosidase SUN41	SUN41	43.7	4.84	1241.07	35.17	1	10	10	27	418
Q9URB4	Fructose-bisphosphate aldolase	FBA1	39.2	6.06	1067.46	67.41	2	19	19	26	359
P83779	Pyruvate decarboxylase	PDC11	62.4	5.58	982.56	46.03	4	19	19	25	567
Q59XX2	Cell surface mannoprotein MP65	MP65	39.2	5.45	949.90	42.06	1	10	10	19	378
P28877	Plasma membrane ATPase 1	PMA1	97.4	4.96	910.11	29.27	8	12	17	20	895
Q5AB48	Secreted protein RBT4	RBT4	37.4	4.55	862.58	32.40	1	7	7	23	358
P46273	Phosphoglycerate kinase	PGK1	45.2	6.48	856.32	68.82	20	17	26	32	417
Q5AL03	Hyphally regulated cell wall protein 1	HYR1	91.9	4.65	854.52	21.00	2	15	15	23	919
C4YJQ8	Elongation factor 2	EFT2	93.3	6.47	833.40	37.17	13	27	27	32	842
O94049	Acetyl-coenzyme A synthetase 1	ACS1	75.1	6.32	762.17	38.52	6	22	24	28	675
P83784	Heat shock protein SSC1, mitochondrial	SSC1	69.7	5.60	735.37	41.20	3	23	23	24	648
Q5AMT2	Glucan 1,3-beta-glucosidase BGL2	BGL2	33.6	4.75	695.24	66.88	2	11	11	21	308
P43098	Fatty acid synthase subunit alpha	FAS2	207.5	5.62	687.01	14.96	4	23	23	24	1885
P14235	Actin	ACT1	41.7	5.69	685.90	68.88	27	11	20	26	376
O13287	6-phosphogluconate dehydrogenase, decarboxylating	DOR14	56.9	6.57	679.44	43.91	5	17	17	20	517
Q5AF03	Glyoxalase 3	GLX3	25.8	4.83	667.99	66.95	1	14	14	19	236
P0CY35	Elongation factor 1-alpha 1	TEF1	50.0	9.03	652.88	39.52	24	6	14	24	458
P87206	ATP-dependent RNA helicase eIF4A	TIF1	44.6	5.36	631.32	55.92	40	17	17	21	397
Q9P8W1	Lipase 4	LIP4	49.5	6.18	616.53	37.47	3	15	15	18	459
Q59TP1	Cell wall protein RBT1	RBT1	73.6	4.34	601.12	13.59	1	8	8	11	721
P46614	Pyruvate kinase	CDC19	55.4	6.99	584.81	48.02	13	17	17	18	504
P29717	Glucan 1,3-beta-glucosidase	XOG1	50.0	5.64	575.69	41.10	1	12	12	17	438
P87222	Ribosome-associated molecular chaperone SSB1	SSB1	66.4	5.38	569.16	40.46	11	17	17	19	613
P83781	Mitochondrial outer membrane protein porin	POR1	29.7	8.57	560.61	57.45	1	16	16	17	282
O74261	Heat shock protein 60, mitochondrial	HSP60	60.1	5.30	529.48	39.40	6	17	17	17	566
P83783	Adenosylhomocysteinase	SAH1	49.0	5.59	524.85	38.44	4	16	16	20	450
Q5AJY5	1,3-beta-glucanosyltransferase PGA4	PGA4	49.0	4.74	523.57	26.83	1	10	10	12	451
P34731	Fatty acid synthase subunit beta	FAS1	227.8	6.02	499.17	13.25	2	20	20	20	2037
Q5AD07	Cell surface Cu-only superoxide dismutase 5	SOD5	23.6	6.87	498.26	29.82	1	5	5	18	228
O94039	Transketolase 1	TKT1	73.7	5.82	486.75	28.66	1	14	14	16	677
Q9P940	Triosephosphate isomerase	TPI1	26.6	6.01	481.44	69.76	3	12	12	16	248
Q5AIR7	Glucan endo-1,3-beta-D-glucosidase 1	ENG1	124.0	5.40	462.03	14.24	2	14	14	14	1145
Q5AFA2	Extracellular glycosidase CRH11	CRH11	46.7	4.96	461.19	26.27	1	14	14	20	453
Q96VB9	Heat shock protein homolog SSE1	MSI3	78.5	5.30	458.97	26.96	6	16	16	19	701

P83776	Hexokinase-2	HXK2	53.4	5.55	447.77	46.69	3	14	16	22	484
A0A1D8PSV5	Small ribosomal subunit protein uS3	RPS3	27.3	9.23	443.58	47.01	2	12	12	15	251
P83778	Malate dehydrogenase, cytoplasmic	MDH1	36.0	5.62	438.56	30.86	1	8	8	10	337
Q9P8Q7	Isocitrate lyase	ICL1	61.4	7.11	414.47	27.27	17	11	11	13	550
P43067	Alcohol dehydrogenase 1	ADH1	36.9	6.44	406.22	42.00	3	12	14	16	350
P83774	Small ribosomal subunit protein RACK1	ASC1	34.5	6.54	383.25	36.28	1	8	8	10	317
P22011	Peptidyl-prolyl cis-trans isomerase	CYP1	17.6	7.97	374.55	34.57	2	6	7	10	162
Q5A8T4	Agglutinin-like protein 1	ALS1	132.8	4.45	372.40	8.57	2	5	6	8	1260
O94038	Alcohol dehydrogenase 2	ADH2	36.8	6.68	358.03	31.90	5	9	11	11	348
P25997	Elongation factor 3	CEF3	116.9	5.73	352.75	17.05	7	14	14	14	1050
Q59M70	NADH-cytochrome b5 reductase 2	MCR1	33.4	8.48	350.38	37.87	2	10	10	11	301
P0CS83	Candidapepsin-2	SAP2	42.3	4.70	342.08	41.96	5	8	8	8	398
P53696	Profilin	PFY1	13.8	5.45	328.68	60.32	1	4	4	6	126
P82610	5-methyltetrahydropteroyltriglutamate--homocysteine methyltransferase	MET6	85.6	5.60	328.26	18.38	2	11	11	11	767
Q5ACR4	Protein OS-9 homolog	YOS9	29.4	5.87	318.31	31.01	1	7	7	8	258
P83775	Putative NADPH-dependent methylglyoxal reductase GRP2	GRP2	37.6	6.44	318.08	34.60	1	10	10	10	341
Q59LS1	Large ribosomal subunit protein uL3	RPL3	43.9	10.26	313.26	24.94	6	9	9	12	389
A0A1D8PCX8	Large ribosomal subunit protein eL6	RPL6	19.8	10.21	300.97	50.00	3	8	8	8	176
O42766	14-3-3 protein homolog	BMH1	29.5	4.81	299.43	39.39	8	10	10	12	264
O13359	Kexin	KEX2	105.1	5.03	292.34	8.42	1	9	9	11	938
P46596	Opaque-phase-specific protein OP4	OPS4	41.3	5.26	287.38	9.70	1	3	3	4	402
C4YG73	Vacuolar protein sorting/targeting protein 10	VPS10	179.2	5.03	284.84	9.27	1	8	11	11	1586
A0A1D8PH52	Acetyl-CoA acetyltransferase	ERG10	41.9	6.90	280.60	27.36	2	9	9	9	402
Q5AKU5	Secreted beta-glucosidase SIM1	SIM1	39.4	4.87	279.75	21.24	1	6	6	7	372
Q5AIA1	Glucan 1,3-beta-glucosidase 2	EXG2	54.5	5.53	268.75	19.83	1	8	8	9	479
P83780	Glucose-6-phosphate isomerase	PGI1	61.1	6.40	267.61	23.45	7	11	11	12	550
P82611	Aconitate hydratase, mitochondrial	ACO1	84.2	6.39	261.09	16.86	8	11	11	12	777
P10875	Tubulin beta chain	TUB2	49.9	4.74	257.32	21.83	13	6	6	7	449
P40953	Chitinase 2	CHT2	60.8	4.89	251.48	12.69	1	4	4	5	583
Q00310	Glycolipid 2-alpha-mannosyltransferase 1	MNT1	50.0	6.90	249.73	31.55	1	9	11	11	431
P22274	ADP-ribosylation factor	ARF1	20.2	5.40	249.10	45.25	1	2	5	7	179
P43071	Multidrug resistance protein CDR1	CDR1	169.8	6.98	238.82	6.26	7	8	8	8	1501
A0A1D8PFG4	Large ribosomal subunit protein eL27	RPL27A	15.5	10.18	238.04	39.71	1	6	6	8	136
A0A1D8PF08	Large ribosomal subunit protein uL2	RPL2	27.3	10.71	237.08	42.91	6	8	8	8	254
A0A1D8PL99	Small ribosomal subunit protein eS6	RPS6A	27.1	10.15	234.92	29.24	3	5	5	5	236

Q8NJN3	Acetyl-coenzyme A synthetase 2	ACS2	73.8	6.13	230.99	13.26	13	8	10	10	671
Q59PT0	V-type proton ATPase subunit B	VMA2	57.2	5.03	230.31	18.75	4	8	8	8	512
Q5AJC0	Extracellular glycosidase UTR2	UTR2	51.7	4.73	228.54	18.30	1	7	7	7	470
P43077	Beta-hexosaminidase	HEX1	63.5	5.59	227.84	12.99	1	6	6	7	562
P0CU34	Peroxiredoxin TSA1-B	TSA1B	21.8	5.06	227.64	45.92	4	7	7	8	196
P83773	Acetyl-CoA hydrolase	ACH1	58.0	6.92	224.35	26.15	3	10	10	11	524
P31225	Corticosteroid-binding protein	CBP1	55.5	5.29	223.02	21.88	1	9	9	9	489
Q59KZ1	Aminopeptidase 2	APE2	104.3	5.36	222.54	10.71	1	8	8	8	924
Q59KI0	UTP--glucose-1-phosphate uridylyltransferase	UGP1	55.5	6.73	221.67	20.00	3	8	8	8	500
O13434	Phosphoenolpyruvate carboxykinase (ATP)	PCK1	60.8	6.60	220.19	22.06	1	10	10	10	553
Q59R28	Alpha-1,2-mannosyltransferase MNN26	MNN26	87.2	6.52	217.91	12.04	1	9	9	9	756
A0A1D8PCW6	Small ribosomal subunit protein uS9	RPS16A	15.7	10.29	214.41	44.37	7	6	6	7	142
Q5AJF7	Large ribosomal subunit protein uL11	RPL12	17.8	9.51	213.45	44.85	4	6	6	6	165
Q07730	Extent of cell elongation protein 1	ECE1	28.9	5.77	209.74	30.26	1	5	5	7	271
A0A1D8PTW6	Hydroxymethylglutaryl-CoA synthase	ERG13	49.7	5.97	207.96	13.97	6	7	7	7	451
P40910	Small ribosomal subunit protein eS1	RPS1	29.0	10.04	203.03	30.08	42	6	6	6	256
C4YKT4	Ras-like protein 1	RAS1	32.2	4.67	201.51	24.31	12	4	4	5	288
Q5ANP2	Nascent polypeptide-associated complex subunit alpha	EGD2	19.5	4.82	201.40	16.85	2	2	2	3	178
A0A1D8PCI6	Small ribosomal subunit protein eS4	RPS42	29.4	10.15	201.06	37.02	5	9	9	10	262
Q5ANA1	Large ribosomal subunit protein eL8B	RPL8B	28.5	10.05	195.96	27.86	5	6	6	6	262
Q5A8I8	Induced during hyphae development protein 1	IHD1	36.9	4.34	192.38	8.42	1	2	2	3	392
Q9UVJ4	Large ribosomal subunit protein uL1	RPL10A	24.4	9.76	191.31	29.95	4	6	6	7	217
Q5A302	Endoplasmic reticulum vesicle protein 25	ERV25	24.5	7.12	188.76	20.00	1	4	4	5	215
A0A1D8PH78	Farnesyl pyrophosphate synthase	ERG20	40.7	4.98	188.63	13.11	3	5	5	6	351
A0A1D8PDT3	Small ribosomal subunit protein uS11	RPS14B	14.0	10.90	186.50	62.88	5	7	7	7	132
A0A1D8PQQ5	Small ribosomal subunit protein uS13	RPS18	17.0	10.35	184.19	35.86	2	5	5	5	145
A0A1D8PLC9	Large ribosomal subunit protein eL20	RPL20B	20.3	10.24	176.57	25.58	1	4	4	5	172
Q5ACI8	Peptidyl-prolyl cis-trans isomerase D	CPR6	40.7	6.39	170.89	13.55	2	3	4	4	369
O42817	Small ribosomal subunit protein uS2	RPS0	28.7	4.91	169.17	24.14	14	5	5	5	261
P53707	37 kDa cell surface protein	CSP37	37.0	7.06	168.09	22.12	1	6	6	6	321
A0A1D8PH21	Large ribosomal subunit protein eL36	RPL36	11.1	11.40	167.93	30.30	2	4	4	5	99
Q59NP1	Copper transport protein CTR1	CTR1	27.8	6.93	167.18	21.91	1	4	4	5	251
P0CY31	Ras-related protein SEC4	SEC4	23.1	5.47	164.22	23.33	1	4	4	5	210
A0A1D8PEY9	Small ribosomal subunit protein eS17	RPS17B	15.7	10.46	163.98	22.63	6	3	3	4	137
A0A1D8PNQ6	Small ribosomal subunit protein eS25	RPS25B	11.6	10.15	163.86	32.38	5	3	3	4	105

A0A1D8PCL1	High-affinity glucose transporter 1	HGT1	60.6	7.62	161.33	10.64	1	6	6	6	545
P83777	Inorganic pyrophosphatase	IPP1	32.1	5.26	159.19	26.39	12	6	6	6	288
A0A1D8PK40	Large ribosomal subunit protein eL19	RPL19A	21.9	11.30	158.04	20.00	1	4	4	5	190
O93827	Mannose-1-phosphate guanyltransferase	MPG1	40.0	6.30	157.66	24.31	15	7	7	7	362
O94072	V-type proton ATPase subunit E	VMA4	25.4	5.40	153.27	21.68	1	4	4	4	226
A0A1D8PDL6	60S ribosomal protein L7-A	RPL7A	27.4	10.24	152.24	32.37	3	6	6	6	241
Q5A7K0	Small ribosomal subunit protein eS24	RPS24	15.5	10.87	152.09	25.19	2	4	4	4	135
Q5AG43	Small ribosomal subunit protein uS7	RPS5	25.3	8.70	150.93	23.11	2	5	5	5	225
Q5A860	Translationally-controlled tumor protein homolog	TMA19	18.5	4.46	148.93	22.16	1	2	2	2	167
Q59MN0	Vacuolar protein 8	VAC8	63.4	5.07	146.74	6.84	5	3	3	3	585
A0A1D8PFV1	Large ribosomal subunit protein uL4	RPL4B	39.2	10.74	146.55	18.46	3	4	4	5	363
P53698	Cytochrome c	CYC1	12.2	9.66	144.99	32.73	4	4	4	5	110
P83782	Cytochrome b-c1 complex subunit 2, mitochondrial	QCR2	39.5	5.57	137.77	14.71	1	4	4	4	374
A0A1D8PN83	Small ribosomal subunit protein uS17	orf19,4149,1	17.6	10.43	135.30	26.45	3	4	4	5	155
P87066	Tubulin alpha chain	TUB1	49.9	5.06	135.08	14.06	16	6	6	6	448
A0A1D8PK43	Large ribosomal subunit protein eL18	RPL18	20.8	11.80	134.55	25.81	2	4	4	5	186
Q5A0X8	Secreted hemophore CSA2	CSA2	15.1	7.50	132.60	33.33	1	4	4	5	147
Q59T44	Small ribosomal subunit protein eS8	RPS8A	22.7	11.09	128.41	14.08	1	4	4	5	206
P39826	Cell division control protein 3	CDC3	47.8	6.64	126.99	9.86	3	4	4	4	416
P0CY33	Cell division control protein 42 homolog	CDC42	21.2	6.54	126.89	15.18	3	3	3	3	191
P52495	Ubiquitin-activating enzyme E1 1	UBA1	114.2	5.01	125.83	8.42	1	6	6	6	1021
O13289	Peroxisomal catalase	CAT1	54.8	6.65	123.48	6.39	3	3	3	3	485
Q96W54	Small ribosomal subunit protein uS8A	RPS22A	14.8	9.88	121.40	31.54	8	4	4	4	130
P82612	Phosphoglycerate mutase	GPM1	27.4	6.16	119.85	20.97	1	5	5	5	248
A0A1D8PGY0	Large ribosomal subunit protein eL21	RPL21A	18.0	10.33	118.85	16.25	5	3	3	3	160
Q59SU1	Candidapepsin-9	SAP9	58.4	5.25	116.66	8.82	2	5	5	5	544
O94083	Eukaryotic translation initiation factor 5A	ANB1	17.1	5.05	113.85	37.34	3	4	4	5	158
A0A1D8PCG7	Small ribosomal subunit protein eS21	RPS21B	9.6	8.15	111.92	28.74	2	2	2	2	87
A0A1D8PGY8	Small ribosomal subunit protein uS4	RPS9B	21.7	10.20	111.53	26.46	5	4	5	5	189
A0A1D8PHH4	Large ribosomal subunit protein eL33	RPL33A	12.1	10.78	111.43	17.76	1	1	1	2	107
Q5AEN2	Large ribosomal subunit protein uL6	RPL9B	21.7	9.51	110.40	22.51	1	4	4	4	191
Q59Y31	Yeast-form wall Protein 1	YWP1	54.2	4.81	109.40	4.50	1	2	2	3	533
P78590	Elongation factor 1-beta	EFB1	23.5	4.40	108.90	19.25	1	2	2	2	213
Q5AJ93	Small ribosomal subunit protein eS7	RPS7A	21.2	9.92	105.10	27.42	1	4	4	4	186
Q5AFN8	Covalently-linked cell wall protein 14	SSR1	22.5	4.68	104.88	5.98	1	1	1	2	234

O13401	Superoxide dismutase [Mn], mitochondrial	SOD2	26.2	8.73	104.17	13.68	1	2	2	2	234
Q5AIB8	Large ribosomal subunit protein uL16	RPL10	25.2	10.08	101.80	15.91	2	3	3	3	220
A0A1D8PK61	Small ribosomal subunit protein eS19	RPS19A	16.1	9.42	100.69	20.00	1	3	3	3	145
Q5A4M8	Protein SUR7	SUR7	29.9	7.69	100.36	32.59	1	5	5	5	270
Q5A4Q1	Adenylate kinase	ADK1	27.6	8.10	98.69	13.25	12	3	3	3	249
Q9UVX1	Lysophospholipase 3	PLB3	81.4	4.84	97.33	4.11	1	3	3	3	754
Q5APD4	Sphingolipid C9-methyltransferase	MTS1	58.7	6.92	96.32	6.04	1	3	3	3	513
P46592	Glycolipid 2-alpha-mannosyltransferase 2	MNT2	54.5	6.67	96.30	12.15	1	5	7	7	461
Q5A900	Small ribosomal subunit protein uS5	RPS21	26.9	10.24	94.70	28.92	1	6	6	6	249
Q5AEN1	Cytochrome c peroxidase, mitochondrial	CCP1	40.7	6.34	94.54	19.13	1	5	5	5	366
Q5A455	Protein transport protein SEC23	SEC23	85.6	5.71	89.83	2.36	1	1	1	1	762
A0A1D8PFL9	Large ribosomal subunit protein eL14	RPL14	14.7	10.90	88.14	24.43	1	3	3	3	131
Q5AB87	Large ribosomal subunit protein uL13	RPL16A	22.6	10.32	87.48	19.50	1	4	4	4	200
Q5ANE3	Non-classical export protein 102	NCE102	18.1	9.01	86.74	12.94	1	1	1	1	170
O42825	GTP-binding protein RHO1	RHO1	22.0	5.73	86.32	16.16	1	2	2	2	198
A0A1D8PHW1	Large ribosomal subunit protein uL5	RPL11	19.8	9.95	84.91	22.99	4	4	4	4	174
P31353	Phosphomannomutase	PMM1	29.0	5.69	81.46	15.08	3	3	3	3	252
A0A1D8PDU3	Small ribosomal subunit protein uS12	RPS23A	16.0	10.81	81.14	12.41	2	2	2	2	145
Q9P926	Nuclear transport factor 2	NTF2	14.2	4.63	79.91	19.35	5	2	2	2	124
Q5AGC4	Cell surface GPI-anchored protein ECM33	ECM331	43.7	4.86	77.06	5.81	1	2	2	3	413
A0A1D8PPN6	Large ribosomal subunit protein eL32	RPL32	14.9	10.54	76.39	24.43	1	3	3	3	131
P40954	Chitinase 3	CHT3	60.0	4.91	76.13	8.11	1	3	3	3	567
O74660	Agglutinin-like protein 4 (Fragments)	ALS4	90.1	4.60	76.06	1.71	2	1	1	1	875
Q5AAU5	Cell wall acid trehalase ATC1	ATC1	120.2	5.21	75.94	4.36	1	3	3	3	1078
P39827	Cell division control protein 10	CDC10	40.7	7.11	75.75	7.28	1	2	2	2	357
Q8TGH6	Guanosine-diphosphatase	GDA1	65.9	5.94	74.94	3.67	1	2	2	2	599
A0A1D8PQN0	Small ribosomal subunit protein eS28	RPS28B	7.5	10.36	74.69	23.88	6	2	2	2	67
Q5A6R1	Large ribosomal subunit protein eL15	RPL15A	24.3	11.34	74.26	22.06	3	4	4	4	204
A0A1D8PPE0	Small ribosomal subunit protein uS15	RPS13	16.9	10.23	72.69	20.53	3	3	3	3	151
Q5AI15	Polyadenylate-binding protein, cytoplasmic and nuclear	PAB1	70.4	5.29	72.50	6.36	3	4	4	4	629
Q5ABP8	Protein ROT1	ROT1	29.9	7.80	72.22	8.46	1	2	2	2	260
Q5AHH4	Small heat shock protein 21	HSP21	21.5	5.35	71.22	14.29	1	3	3	4	189
Q59S78	Small COPII coat GTPase SAR1	SAR1	21.5	5.59	69.06	17.89	26	3	3	3	190
O94200	ATP-dependent 6-phosphofructokinase subunit beta	PFK2	104.0	6.35	68.76	5.39	1	4	4	5	946
Q5A389	Small ribosomal subunit protein uS10	RPS20	13.3	9.94	68.18	16.81	1	2	2	2	119

O74226	Cell wall synthesis protein KRE9	KRE9	29.1	8.18	68.04	10.33	1	2	2	2	271
A0A1D8PM41	Large ribosomal subunit protein eL22	RPL22B	14.1	5.36	67.50	17.74	1	1	1	1	124
A0A1D8PM75	Large ribosomal subunit protein eL30	RPL30	11.5	9.70	64.04	21.70	6	2	2	2	106
Q5AJB1	V-type proton ATPase catalytic subunit A	TFP1	67.6	5.27	63.55	8.27	6	4	4	4	617
A0A1D8PHF5	Large ribosomal subunit protein eL31	RPL31B	13.0	9.82	63.10	19.64	1	2	2	2	112
Q96UX5	Heat shock protein 78, mitochondrial	HSP78	91.6	6.80	62.03	1.97	2	1	2	2	812
Q59P03	NADH-cytochrome b5 reductase 1	CBR1	32.5	8.10	60.68	6.12	1	2	2	3	294
P46250	SEC14 cytosolic factor	SEC14	34.7	6.40	60.33	5.65	1	2	2	2	301
A0A1D8PTI7	Small ribosomal subunit protein eS27	RPS27	9.0	8.73	60.08	28.05	1	2	2	2	82
Q5AKA5	Cys-Gly metallodipeptidase DUG1	DUG1	53.6	5.24	58.30	4.33	1	2	2	2	485
A0A1D8PK22	Small ribosomal subunit protein uS19	RPS15	15.9	10.32	55.23	17.61	1	2	2	2	142
Q9P8V9	Lipase 8	LIP8	49.7	5.49	55.22	2.17	1	1	1	1	460
Q59WB3	S-adenosylmethionine permease GAP4	GAP4	66.4	7.46	54.40	1.98	1	1	1	1	607
Q59VR3	FK506-binding protein 3	FPR3	47.6	4.46	54.32	3.29	1	1	1	1	426
Q5AP65	Protein FMP52, mitochondrial	FMP52	24.3	8.98	53.22	21.83	1	5	5	5	229
Q5ADQ6	Small ribosomal subunit protein eS12	RPS12	15.7	4.70	52.68	23.08	1	3	3	3	143
O13425	Serine hydroxymethyltransferase, mitochondrial	SHM1	54.5	8.97	50.91	6.49	4	3	3	3	493
Q59V93	Very-long-chain 3-oxoacyl-CoA reductase	CAALFM_CR06070WA	38.3	9.57	50.21	3.15	1	1	1	1	349
Q5A6A1	Large ribosomal subunit protein eL24	RPL24A	17.4	11.37	50.20	12.26	3	2	2	2	155
Q5AP66	Phosphatidylinositol transfer protein SFH5	SFH5	36.6	5.33	50.06	3.75	1	1	1	1	320
P43060	Phosphoribosylaminoimidazole-succinocarboxamide synthase	ADE1	32.9	5.50	49.23	14.43	3	3	3	3	291
Q5AGZ7	Large ribosomal subunit protein uL18	RPL5	34.5	7.64	48.82	8.05	2	2	2	2	298
Q5A477	GDP-mannose transporter	VRG4	41.2	9.36	48.75	2.70	1	1	1	1	371
P53704	Glutamine--fructose-6-phosphate aminotransferase [isomerizing] OS=*Candida albicans* (strain SC5314 / ATCC MYA-2876) OX=237561 GN= PE=1 SV=3 - [GFA1_CANAL]	GFA1	79.2	6.24	48.45	5.89	2	3	3	3	713
A0A1D8PK71	60S ribosomal protein CAALFM_C304810CA	orf19,5943,1	29.1	9.14	48.35	8.24	1	2	2	2	267
Q8X1E6	Hsp90 co-chaperone Cdc37	CDC37	58.5	4.81	47.59	7.09	1	4	4	4	508
P43102	Ubiquitin-conjugating enzyme E2 4	UBC4	16.3	7.40	47.38	4.76	2	1	1	1	147
Q5A651	Candidapepsin-10	SAP10	49.3	4.51	46.68	2.43	1	1	1	1	453
Q59UT4	GPI-anchored hemophore RBT5	RBT5	24.2	4.37	45.36	4.15	2	1	1	1	241
Q59WF4	Alpha-1,2-mannosyltransferase MNN2	MNN2	69.1	6.27	45.18	2.51	1	1	1	1	597
P52498	Ras-related protein RSR1	RSR1	27.6	5.21	44.76	10.48	1	2	2	2	248
O59931	Large ribosomal subunit protein eL13	RPL13	23.0	10.61	44.68	13.86	1	3	3	3	202
O13426	Serine hydroxymethyltransferase, cytosolic	SHM2	52.0	7.20	44.26	3.62	4	2	2	2	470
Q59TE0	Large ribosomal subunit protein uL22	RPL17B	21.0	10.77	43.82	17.84	3	3	3	3	185

P10977	Vacuolar aspartic protease	APR1	45.4	4.83	43.74	5.97	1	1	1	1	419
P78591	Iron transport multicopper oxidase FET3	FET3	70.6	4.92	43.57	1.28	1	1	1	1	624
Q5AAI8	Nucleosome assembly protein 1	NAP1	49.5	4.31	43.55	3.22	1	1	1	1	435
Q92410	Alpha,alpha-trehalose-phosphate synthase [UDP-forming]	TPS1	54.4	6.23	42.82	7.74	1	3	3	3	478
P79023	Phospho-2-dehydro-3-deoxyheptonate aldolase, tyrosine-inhibited	ARO4	40.3	6.65	42.40	4.59	5	2	2	2	370
Q59MV9	Flavohemoprotein	YHB1	45.8	5.83	42.39	3.02	1	1	1	1	398
Q5ALV6	Small ribosomal subunit protein eS26	RPS26A	13.6	10.89	42.24	7.56	1	1	1	2	119
A0A1D8PI15	Small ribosomal subunit protein eS10A	RPS10	13.8	9.83	41.95	11.86	1	1	1	1	118
P34732	Vesicular-fusion protein SEC18	SEC18	88.9	7.43	41.83	2.14	1	1	1	1	794
Q5AFE4	Regulator of cytoskeleton and endocytosis RVS161	RVS161	30.1	7.14	41.71	2.65	1	1	1	1	264
P28870	FK506-binding protein 1	RBP1	13.3	7.18	41.37	12.10	1	1	1	1	124
Q5ABA2	Ceramide-binding protein SVF1	SVF1	43.0	5.07	41.07	6.02	1	2	2	2	382
Q5APC0	Golgi apparatus membrane protein TVP18	TVP18	18.8	7.74	40.37	10.98	1	1	1	1	173
Q9HGT6	Serine--tRNA ligase, cytoplasmic	SES1	53.0	5.73	39.53	5.19	2	2	2	2	462
O42778	Candidapepsin-8	SAP8	43.0	6.35	38.96	7.65	2	2	2	2	405
Q5ANL6	13 kDa ribonucleoprotein-associated protein	SNU13	13.6	7.97	38.81	19.05	2	1	1	1	126
Q5A8K2	Alanine--tRNA ligase	ALA1	108.2	6.07	38.63	2.17	1	2	2	2	969
Q5A8A2	Phosphatidylglycerol/phosphatidylinositol transfer protein	NPC2	21.2	5.05	37.66	4.17	1	1	1	1	192
P43065	Saccharopine dehydrogenase [NAD(+), L-lysine-forming]	LYS1	42.4	5.44	37.12	2.62	1	1	1	1	382
Q5A2J7	Calcium channel YVC1	YVC1	77.3	5.08	36.59	1.48	1	1	1	2	675
O93852	D-arabinono-1,4-lactone oxidase	ALO1	63.4	6.61	36.44	3.59	1	2	2	2	557
O74254	Glucoamylase 1	GAM1	105.7	4.92	35.37	1.90	1	1	1	1	946
Q5AD51	Ergosterol biosynthetic protein 28	ERG28	16.0	9.31	35.05	7.97	1	1	1	1	138
A0A1D8PTR4	Small ribosomal subunit protein uS14	CAALFM_CR08480CA	6.6	9.76	34.88	12.50	2	1	1	1	56
Q59YF0	Protein transport protein SSO2	SSO2	34.3	5.36	34.81	3.05	1	1	1	1	295
A0A1D8PCQ5	Large ribosomal subunit protein uL24	RPL26B	14.2	10.48	34.72	7.09	3	1	1	1	127
P0CY19	Deoxyuridine 5'-triphosphate nucleotidohydrolase	DUT1	16.9	5.92	33.06	5.03	1	1	1	1	159
Q5A5S7	Autophagy-related protein 27	ATG27	28.3	5.53	33.05	2.78	1	1	1	1	252
A0A1D8PI71	Squalene synthase ERG9	ERG9	51.2	6.84	32.14	2.01	2	1	1	1	448
Q5ABU8	Beta-mannosyltransferase 6	BMT6	74.5	7.72	31.42	1.08	1	1	1	1	646
A0A1D8PPT5	Large ribosomal subunit protein uL14	RPL23A	14.5	10.24	31.17	5.84	1	1	1	1	137
Q5AI86	Eukaryotic translation initiation factor 3 subunit I	TIF34	38.2	5.47	31.01	4.00	1	1	1	1	350
Q5AAU3	Protein transport protein SEC31	PGA63	136.2	6.77	30.10	1.34	1	1	1	1	1265
Q59X23	Dolichyl-phosphate-mannose--protein mannosyltransferase 4	PMT4	86.6	8.87	30.05	1.06	1	1	1	1	755
Q5A846	Beta-mannosyltransferase 3	BMT3	62.9	6.89	30.01	1.64	1	1	1	1	549

C4YKP5	Increased recombination centers protein 22-1	IRC22	25.9	5.68	29.99	5.02	4	1	1	1	239
Q5ACZ2	Mannan endo-1,6-alpha-mannosidase DFG5	DFG5	50.0	4.65	29.78	1.77	1	1	1	1	451
Q59W44	Mitochondrial import inner membrane translocase subunit TIM50	TIM50	54.2	6.65	29.70	2.99	1	1	1	1	469
P87024	Beta-glucan synthesis-associated protein SKN1	SKN1	83.7	5.15	28.63	2.44	1	1	1	1	737
C4YQD9	S-methyl-5'-thioadenosine phosphorylase	MEU1	37.5	7.62	28.46	3.20	2	1	1	1	344
P46586	ATP phosphoribosyltransferase	HIS1	32.6	5.34	28.44	5.37	1	1	1	1	298
A0A1D8PLI2	Isopentenyl-diphosphate delta-isomerase	IDI1	32.3	4.97	28.32	2.82	1	1	1	1	284
A0A1D8PPS1	Large ribosomal subunit protein uL23	RPL25	15.8	10.15	27.95	10.56	2	1	1	1	142
Q5ADQ9	Beta-mannosyltransferase 1	BMT1	79.6	7.88	27.05	2.92	1	2	2	2	684
Q5AD78	Mannan endo-1,6-alpha-mannosidase DCW1	DCW1	50.3	4.73	26.94	2.21	1	1	1	1	452
A0A1D8PDT4	Large ribosomal subunit protein eL39	RPL39	6.3	12.31	25.51	19.61	4	1	1	1	51
Q59NX9	Diphthine methyl ester synthase 1	DPH5	33.9	4.87	25.38	6.35	4	2	2	2	299
P34948	Mannose-6-phosphate isomerase	PMI1	48.8	5.33	25.06	7.26	1	2	2	2	441
O43101	H/ACA ribonucleoprotein complex subunit CBF5	CBF5	54.3	9.20	24.94	2.09	1	1	1	1	479
Q59Q46	Inosine-5'-monophosphate dehydrogenase	IMH3	56.2	6.55	23.48	2.30	1	1	1	1	521
A0A1D8PP14	Large ribosomal subunit protein eL43	RPL43A	10.1	10.68	23.21	9.78	1	1	1	1	92
A0A1D8PC43	Diphosphomevalonate decarboxylase	MVD	39.5	6.46	21.01	3.04	1	1	1	1	362
Q5A893	F-actin-capping protein subunit alpha	CAP01	32.0	5.52	20.89	5.71	1	1	1	1	280
